# Ubiquitous anaerobic ammonium oxidation in inland waters of China: an overlooked nitrous oxide mitigation process

**DOI:** 10.1038/srep17306

**Published:** 2015-11-27

**Authors:** Guibing Zhu, Shanyun Wang, Leiliu Zhou, Yu Wang, Siyan Zhao, Chao Xia, Weidong Wang, Rong Zhou, Chaoxu Wang, Mike S. M. Jetten, Mariet M. Hefting, Chengqing Yin, Jiuhui Qu

**Affiliations:** 1 Key Laboratory of Drinking Water Science and Technology, Research Center for Eco-Environmental Sciences, Chinese Academy of Sciences, Beijing 100085, China; 2 Department of Microbiology, Radboud University Nijmegen, the Netherlands; 3 Ecology and Biodiversity Group, Department of Biology, Utrecht University, the Netherlands; 4 Department of Biogeochemistry, Max Planck Institute for Marine Microbiology, Bremen, Germany

## Abstract

Denitrification has long been regarded as the only pathway for terrestrial nitrogen (N) loss to the atmosphere. Here we demonstrate that large-scale anaerobic ammonium oxidation (anammox), an overlooked N loss process alternative to denitrification which bypasses nitrous oxide (N_2_O), is ubiquitous in inland waters of China and contributes significantly to N loss. Anammox rates in aquatic systems show different levels (1.0–975.9 μmol N m^−2^ h^−1^, *n* = 256) with hotspots occurring at oxic-anoxic interfaces and harboring distinct biogeochemical and biogeographical features. Extrapolation of these results to the China-national level shows that anammox could contribute about 2.0 Tg N yr^−1^, which equals averagely 11.4% of the total N loss from China’s inland waters. Our results indicate that a significant amount of the nitrogen lost from inland waters bypasses denitrification, which is important for constructing more accurate climate models and may significantly reduce potential N_2_O emission risk at a large scale.

For decades, denitrification by heterotrophic bacteria was assumed to be the only pathway for loss of fixed nitrogen to the atmosphere^1^. The discovery of anaerobic ammonium oxidation (anammox) mediated by autotrophic anammox bacteria oxidizing ammonia directly to nitrogen gas (N_2_) without emission of nitrous oxide (N_2_O) challenged this view^2^,^3^,^4^. So far, extensive large-scale occurrence of anammox has been detected in marine ecosystems and makes a significant contribution to N loss^5^,^6^. In terrestrial ecosystem, however, the large-scale occurrence of anammox in inland waters, and how much anammox in these systems influences the global nitrogen cycle, is not yet known^7^,^8^,^9^,^10^.

The oxic–anoxic interface has long been regarded as the hotspot for biogeochemical cycles, owing to extensive interactions of aerobic and anaerobic microorganisms spanning from microscale to macroscale^11^. Marine anammox hotspots occur in oxygen minimum zones (OMZ) which are oxic–anoxic interfaces with intensive material exchange, in particular of oxidized (NO_x_^−^) and reduced (NH_4_^+^) nitrogen compounds^12^,^13^,^14^,^15^, providing the substrate for anammox bacteria. In terrestrial ecosystems, anammox hotspots have been identified at land-freshwater interfaces in riparian zones^7^, another oxic–anoxic interface with intensive exchange of nitrogen compounds^9^. Hence we hypothesize, based on previous research^16^,^17^,^18^,^19^, that large-scale anammox occurs at oxic–anoxic interfaces in various inland waters and wetlands.

The oxic–anoxic interfaces of various inland waters and wetlands, including soil–water and sediment–water interfaces, play important roles in regulating landscape-level interaction in vertical, transversal and longitudinal dimension^20^,^21^. The present study aimed to investigate the extensive occurrence and biogeochemical features of anammox in inland waters and wetlands and to determine the qualitative and quantitative significance of nitrogen loss via anammox. The hypothesis that extensive anammox occurs at the oxic–anoxic interface of inland waters was first tested in different types of inland waters, and then further confirmed by nationwide sampling. Environmental factors contributing to the mitigation of N_2_O emission were also investigated.

## Results

### Anammox at oxic–anoxic interfaces

**Figure 1:**
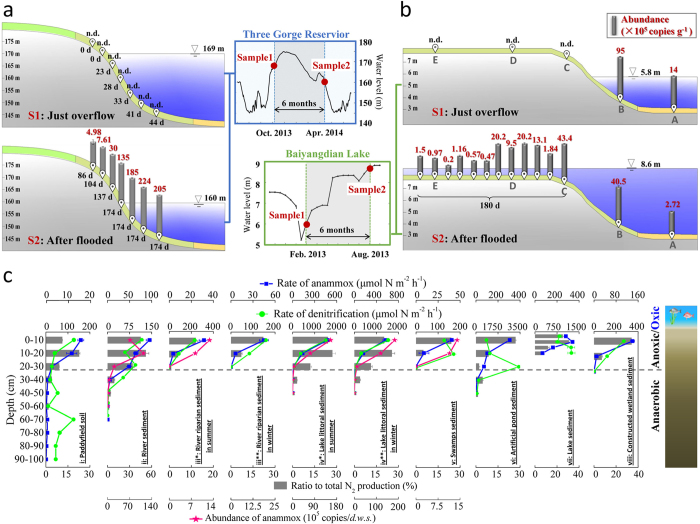
The anammox process at oxic-anoxic interfaces of inland waters. Anammox occurred at water-soil interface in the Three Gorge Reservior (***a***) and Baiyangdian Lake (***b***) along with water flooding duration indicated by the anammox abundance variation and the water level fluctuation. n.d. indicates an abundance value below the detection limit (<10^3^). Fluctuations of water level, flooding duration of sampling sites and sampling time in the Three Gorges Reservoir and Baiyangdian Lake are also shown in plots, respectively. (***c***) Anammox at water-sediment interfaces mainly occurred in surface and subsurface sediments and soils (0–40 cm).

The oxic–anoxic interfaces of inland waters, mainly comprising soil–water and sediment–water interfaces in riparian zones and sediment–water interfaces in open waters, were selected for this study (Fig. 1).

#### Soil-water interface

At the soil-water interface, polymerase chain reaction (PCR) screening showed negative results from dry soils in the riparian zone in Three Gorges Reservoir (*n *= 9) (Fig. 1a and Supplementary Fig. 1) and in Baiyangdian Lake (*n *= 9) (Fig. 1b and Supplementary Fig. 2), with a detection limit of around 1.00 × 10^3^ gene copies g^−1^ dry soil. More interestingly, after flooding for few months anammox bacteria were detected in every sample from the same locations. In the Three Gorges Reservoir, quantitative PCR assays targeting the hydrazine synthase (*hzs*) gene which is specific for anammox, showed that the anammox bacteria were detectable in every sample (*n *= 30) after flooding for more than three months and their abundance increased from landward ((4.98 ± 2.39) × 10^5^ copies g^−1^ dry soil) to waterward ((2.24 ± 0.2) × 10^6^ copies g^−1^ dry soil) along with the flooding duration at rates of (0.6−11.8) × 10^4^ copies g^−1^ d^−1^. In Baiyangdian Lake, after flooding for six months the anammox bacteria were also all detectable in every sample (*n = *36), and the abundance ranged from (1.97 ± 0.1) × 10^4^ to (4.34 ± 0.2) × 10^6^ copies g^−1^ with rates of (0.2–26.3) × 10^3^ copies g^−1^ d^−1^. The anammox bacteria are not likely to originate from the water column because no positive PCR results were obtained from water column samples, or from soils flooded for less than one month. The above indicates anammox to occur ubiquitously in water-covered area.

#### Sediment–water interface

The results above motivated us to characterize and quantify anammox in the sediments of inland waters. The sediment cores samples (*n *= 10) from sediment–water interfaces in the Three Gorges Reservoir, Baiyangdian Lake, and other inland waters with different nitrogen loadings were investigated to determine whether and how much anammox occurred and contributed using ^15^N isotope tracing and molecular methods (Supplementary Fig. 3). The nitrogen isotope results showed that active anammox was detected in every sample, and mainly occurred at the surface of and in the subsurface of sediments (0–40 cm) (Fig. 1c and Supplementary Fig. 4). Quantitative PCR assays showed that anammox bacterial abundance mainly distributed within 50 cm below the surface.

### Ubiquity of anammox in inland waters

**Figure 2:**
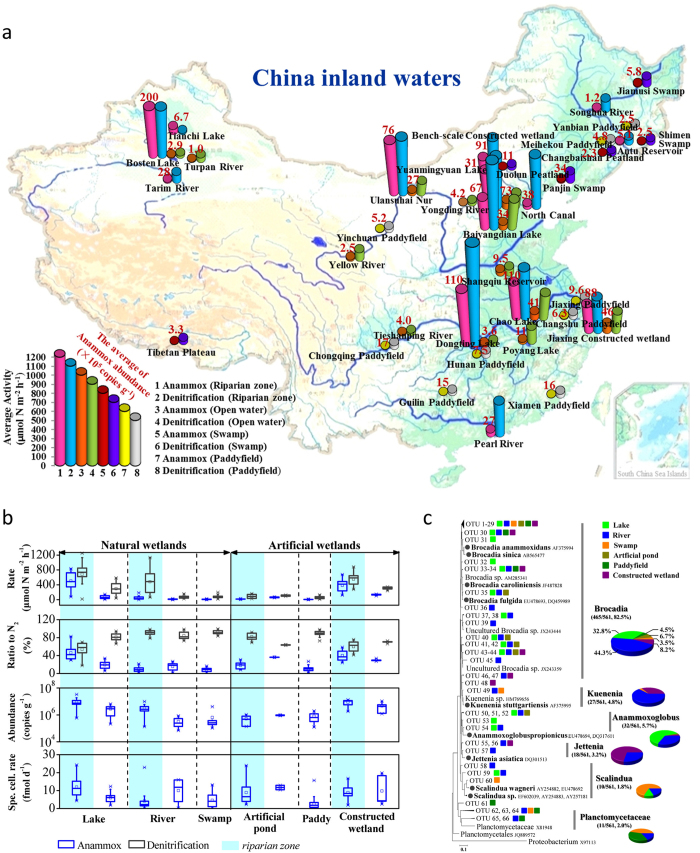
The ubiquitous and large-scale anammox process in Chinese inland waters. (***a***) biogeographical distribution of anammox bacterial abundance with anammox and denitrification rates in inland waters and wetland ecosystems from latitude 22 to 44 degrees north, longitude 80 to 122 degrees west in Chinese territory; (***b***) statistical analysis of anammox bacterial rates, abundance, contribution, and specific cellular rates in various inland water ecosystems; (***c***) anammox bacterial population community in various inland water ecosystems. Phylogeny of the anammox sequences from Chinese inland waters constructed by neighbor-joining method using Kimura two-parameter distance with 1000 bootstrap in the MEGA 4.0 package, the DOTUR program was used with 3% sequence variation for OTU determination. The map derives from the web version of “Data Sharing Infrastructure of Earth System Science” http://www.geodata.cn. All of the maps used in the manuscript are free. With the map we use the EXCEL software to draw the column or pie at the same bar scale and paste them on the sampling site in the map to create the figure.

We increased the range of sampling sites to the national scale to investigate the occurrence and importance of anammox in inland waters. A total of 256 sediment/soil core samples (0–50 cm) from oxic–anoxic interfaces of inland waters were investigated and covered a wide range of climatic zones, spanning from northern latitude 22 to 44 degrees and eastern longitude 80 to 120 degrees (Fig. 2 and Supplementary Table S1, S2).

Screening of 16S rRNA gene sequences revealed that anammox bacteria were ubiquitous in all of the investigated inland waters, including rivers (*n *= 22), river riparian zones (*n *= 29), lakes (*n *= 26), lake riparian zones (*n *= 30), paddy fields (*n *= 65), reservoirs (*n *= 15), peatlands (*n *= 11), swamps (*n *= 30) and constructed wetlands (*n *= 28). Quantitative PCR assays showed that anammox bacterial abundance ranged from 3.1 × 10^4^ to 3.3 × 10^7^ copies g^−1^, with lake riparian zones harboring the highest abundance (*P *= 0.000) (Fig. 2a,b and Supplementary Fig. 5). Phylogenetic analysis identified all known anammox bacterial species, with *Brocadia* and *Kuenenia* as the dominant species among 610 anammox 16S rRNA gene sequences analyzed (Fig. 2c). The anammox bacterial biodiversity in inland waters was much higher than that in marine ecosystems^22^.

Anammox rates were measured by the ^15^N isotope tracing method in intact core samples. Anammox occurred in every sample at rates ranging from 1.0 to 975.9 μmol N m^−2^ h^−1^ (*n *= 256), corresponding to 0.9–82.2% of the total N loss with high heterogeneity (Fig. 2b). Strikingly in some samples, the anammox rates for N_2_ production were even higher than those of denitrification (*n *= 12). These data indicate that denitrification is not the only significant pathway for N loss to the atmosphere from inland waters.

The ubiquitous occurrence of anammox in inland waters could be reflective of special ecophysiological features of anammox bacteria. Anammox bacteria have a very low half-saturation constant (*K*_*s*_) value for substrate ammonia (<5 μM)^23^ and substrate nitrite (0.2–0.3 μM for *Candidatus* Kuenenia and <5 μM for *Candidatus* Brocadia)^24^, which may give the anammox bacteria a selective advantage in competing for substrates with either ammonia oxidizing bacteria (*K*_*s*_ of 0.14 mM NH_4_^+^ for *Nitrosospira* sp. *AV* and 1.9 mM NH_4_^+^ for *Nitrosomonas europaea*, the two most extensively studied)^25^ or nitrite oxidizing bacteria (*K*_*s*_ of 0.01 mM for ‘*Nitrospira*’ and 0.02–0.14 mM for ‘*Nitrobacter*’, the two most important species)^26^. Furthermore, anammox bacteria harbor the functional combination of two specific anammox structures, (i) the anammoxosome membrane of ladderane lipids, a dense and low permeability membrane, which could maintain concentration gradients during the exceptionally slow anammox metabolism^27^, and (ii) key transporters of ammonium (*Amt*) and nitrite (*FocA*, *NarK*)^28^. These special features of anammox bacteria contribute to the widespread occurrence of anammox in various kinds of inland waters and extreme environments.

### Features and significance of anammox in inland waters

**Figure 3:**
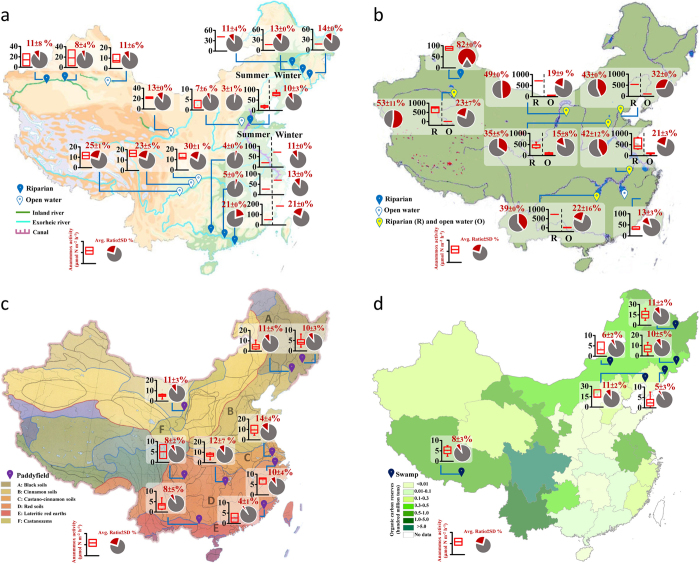
Biogeographical distribution of anammox (red) and denitrification (grey) rates and their contribution to the N loss in sole inland waters including rivers (*a*), lakes (*b*), paddy fields (*c*) and swamps (*d*). Box charts (the horizontal line indicates the median, box gives the 25^th^ and 75^th^ percentiles, and whisker shows range from the 5^th^ to 95^th^ percentile) represent anammox bacterial activity, and pie charts represent the contribution of anammox (red) and denitrification (grey) to the total N loss. The map were come from web of “Data Sharing Infrastructure of Earth System Science” http://www.geodata.cn. All of the maps used in the manuscript are free. With the map we use the EXCEL software to draw the column or pie at the same bar scale and paste them on the sampling site in the map to create the figure.

The high spatiotemporal heterogeneity of anammox rates in inland waters on a national scale prompted us to investigate the biogeochemical and biogeographical features of anammox in particular aquatic ecosystems (Fig. 3).

#### Rivers

Results from river ecosystems, including exorheic rivers, inland rivers and canals, showed that the anammox rate in open water sediments (6.9–15.4 μmol N m^−2^ h^−1^, *n *= 15) was lower than that in riparian zone sediments (17.1–70.9 μmol N m^−2^ h^−1^, *n *= 23) (*p *= 0.000) (Fig. 3a). Along the river path, anammox rates did not show much variation with sampling distances <5 km either in riparian zone or open waters. However, in estuary zones, such as the mouth of the Pearl River, the anammox rate significantly increased from 25.3–70.4 μmol N m^−2^ h^−1^ (4.9–11.2% to total N loss, *n *= 4) to 83.2–149.4 μmol N m^−2^ h^−1^ (20.8–21.2%, *n *= 2). The contribution of anammox to N loss in river ecosystems in China was estimated to be (1.1 ± 0.7) × 10^5^ t N yr^−1^, based on statistical analysis of anammox rates in riparian zones and open waters in different seasons.

#### Lakes

Among the tested aquatic ecosystems, the highest anammox rates (348.1–719.6 μmol N m^−2^ h^−1^, *n *= 24) were recorded in the sediments of lake riparian zones, and were much higher than those in other aquatic ecosystems (6.7–56.1 μmol N m^−2^ h^−1^, *n *= 232) (*p *= 0.000) (Fig. 3b). Moreover, the anammox rates in lake riparian zones were one order of magnitude higher than those in open water (30.3–107.4 μmol N m^−2^ h^−1^, *n *= 22), showing great spatial heterogeneity of the anammox process in lake ecosystems compared with rivers. The highest numbers of anammox cells were also detected in the interface sediments of lake riparian zones (6.4 × 10^6^–1.2 × 10^7^ copies g^−1^, *n *= 24) compared with other inland waters (2.2 × 10^5^–1.23 × 10^6^ copies g^−1^, *n *= 232) (*p *= 0.000) (Fig. 2b). Moreover, high specific cellular anammox activities (7.7–15.0 fmol day^−1^, *n *= 24), around the upper end of reported values (2–20 fmol d^−1^)^2^,^29^, were observed in the sediments of lake riparian zones. These results indicate that lake riparian zones are a hotspot for anammox processes among inland waters. Consequently, anammox in lake ecosystems played a considerable role in N loss, (5.6 ± 3.5) × 10^5^ t N yr^−1^, based on statistical analysis of anammox rates in lake riparian zones and open waters.

The notion that lakes, rather than rivers, hold anammox hotspots is somewhat in disagreement with our previous assumption that river riparian zones might be the principal anammox centers owing to high ammonia flux in rivers, derived from the terrestrial soil surface^30^. The possible reason for the higher anammox activities in lake littoral zones could be their hydrological regimes. The long hydraulic retention time in lake riparian zones would provide the efficient biomass aggregation necessary for anammox bacteria as slow growers. Furthermore, the vertical water-level fluctuation would enhance the exchange of nitrogen compounds in water with riparian zones, and thus provide nitrite to the anammox process, especially when nitrification is stimulated in this zone on exposure to air with water level fluctuation^8^,^31^,^32^,^33^.

#### Paddy fields

Paddy fields are regarded as one of the most significant nitrogen sinks and significant N_2_O emission sources in terrestrial ecosystems^34^. We investigated paddy fields in China (*n *= 65) with a wide range of soil types, including black soils, cinnamon soils, castano-cinnamon soils, red soils, lateritic red earths and castanozems. Our results showed that anammox was widespread in paddy soil ecosystems, with rates of 3.3–7.8 μmol N m^−2^ h^−1^ and a contribution of 6.7–12.7% to N loss (Fig. 3c), with limited range and heterogeneity. An estimated total N loss of (1.1 ± 0.7) × 10^6^ t N yr^−1^ can be attributed to anammox in Chinese paddy field ecosystems, equivalent to 4.6% of the amount of N chemical fertilizer consumed nationally (2.38 × 10^7^ Mg N in 2011)^35^. This result explains the gap in N loss from paddy fields that could not be attributed to NH_3_ volatilization, N_2_O emission, runoff or leaching^36^. The present study indicates that N cycle analysis of paddy soil ecosystems needs to be augmented with the anammox process.

#### Swamps

Swamp ecosystems, especially peatlands, are rich in carbon. Our research in swamp wetlands showed that anammox ubiquitously occurred in peatlands, moss bogs and meadow marshes, with rates of 3.1–12.4 μmol N m^−2^ h^−1^ and a contribution of 5–11% to N loss (*n *= 41) (Fig. 3d), showing little heterogeneity. Although the anammox rates in swamps are not high, this finding extends our knowledge of anammox habitats from conventional low carbon N-rich, to high carbon N-deficient environments^29^. Combined with the area of swamps, the contribution of anammox in swamps to nitrogen loss was estimated as (8.1±6.6) × 10^4^ t N yr^−1^.

Biogeochemical correlation analysis showed that nitrate contents had the most positive influence on anammox rates in rivers (*r *= 0.695, *P *= 0.000), lakes (*r *= 0.626, *P *= 0.000) and swamps (*r *= 0.438) (Supplementary Table 3). The possible reason for nitrate content as the key factor in determining anammox rate may be that the sampling sites with allochthonous ammonia input were characterized by high ammonia pollution, which was in agreement with literature reports that 10 units of anammox reaction need 10 units of ammonia and nitrate respectively^7^,^29^. Biogeographical correlation analysis identified temperature and altitude as the most positive (*r *= 0.332) and negative (*r *= −0.446) influences on anammox rates (Supplementary Table 4). The positive influence of temperature on anammox has been reported by many researchers^9^,^29^. The negative influence of altitude may be attributable to the lower human activities and allochthonous N pollutant input at high altitudes, such that anammox would be substrate limited (which is also in agreement with the biochemical analysis). Based on the total area of various inland water and wetlands in China, and considering the great spatiotemporal heterogeneity of anammox in rivers and lakes, we estimate that about 2.0 (±0.7) Tg yr^−1^ (equal to (11.4 ± 5.0)%) of N loss may be attributed to the anammox process (Table 1; Supplementary Table 5).

### Anammox and N_2_O flux at oxic–anoxic interfaces

**Figure 4:**
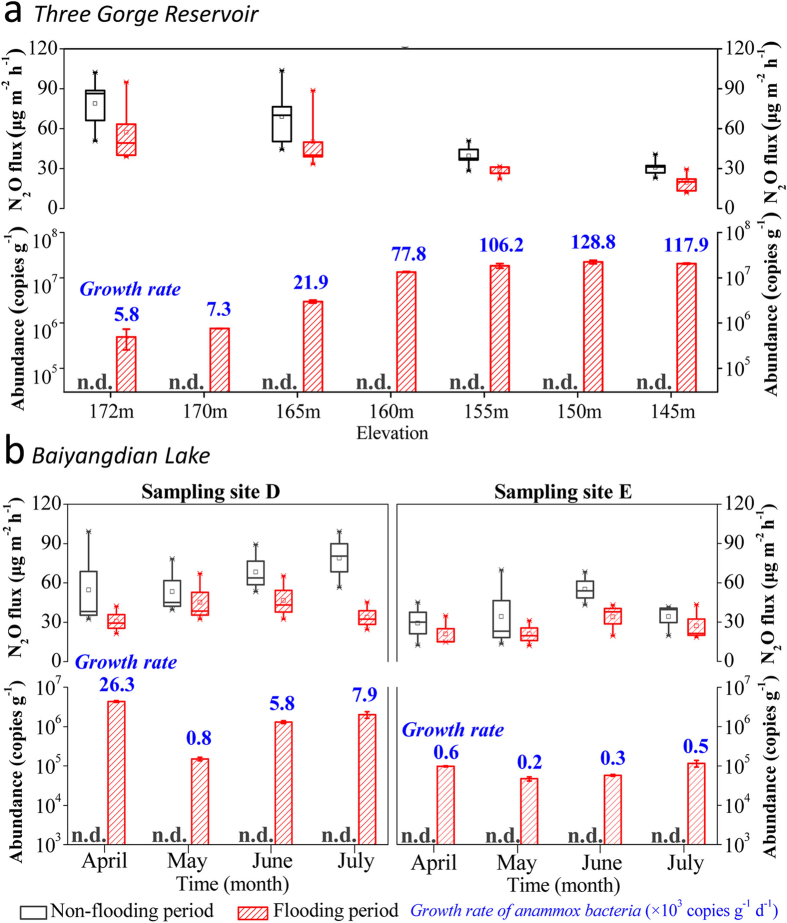
Spatiotemporal variation of anammox abundance and N_2_O flux between non-flooding and flooding period in the Three Gorges Reservoir (*a*) and Baiyangdian Lake (*b*). Error bars indicate *s.d.* (*n* = 3) and n.d. indicates an abundance value below the detection limit (<10^3^). The columns provide anammox abundance, numbers (blue) above columns indicate the anammox bacterial growth rate, and box charts represent the flux of N_2_O at various elevations and time points (horizontal line indicates the median, box gives the 25^th^ and 75^th^ percentiles, and whisker shows range from the 5^th^ to 95^th^ percentile).

The N_2_O fluxes in the riparian zones of the Three Gorges Reservoir and Baiyangdian Lake were measured accompanying with the water level fluctuation, using a closed-chamber technique over a four-season period (Fig. 4).

In the riparian zone of the Three Gorges Reservoir, we found N_2_O fluxes at the soil-water interfaces in the flooding period to be lower than those during the non-flooding period (28.4%, 27.3% and 26.9% lower on average at 175 m, 165 m and 155 m height above sea level, respectively), along with the average increase of anammox bacterial abundance (5.80 × 10^3^ copies g^−1^ d^−1^ of 172 m, 2.19 × 10^4^ copies g^−1^ d^−1^ of 165 m and 1.06 × 10^5^ copies g^−1^ d^−1^ of 155 m) (Fig. 4a). A part of N_2_O will be dissolved in the overlying water (1.5–1.8 mg L^−1^)^37^ during the flooding period, but it is negligible compared with the overall flux variation. At the water-sediment interface (site of 145 m height), the measured N_2_O fluxes were between 11.70 and 29.58 μg m^−2^ h^−1^, with abundant anammox bacteria ((2.24 ± 0.20) × 10^7^ copies g^−1^). These spatiotemporal water-level fluctuation results showed that sites with higher anammox abundance had lower N_2_O emission (*r *= −0.877, *P* = 0.000) (Supplementary Table 6).

In Baiyangdian Lake, we also found that the N_2_O flux decreased from 44.39–78.85 μg m^−2^ h^−1^ (site D in Fig. 1b, *n *= 20) and 31.58–43.83 μg m^−2^ h^−1^ (site E in Fig. 1b, *n *= 20) before the flooding, to 22.60–46.96 μg m^−2^ h^−1^ (site D, *n *= 20) and 17.88–33.03 μg m^−2^ h^−1^ (site E, *n *= 20) after flooding, respectively (Fig. 4b). In our previous study, it was clear that substantial anammox activity could mitigate undesirable N_2_O emissions^17^. We propose that sites undertaking a substantial N loss via the anammox pathway will have lower N_2_O emission rates than sites where denitrification dominates. Although other environmental variations, such as microbial nitrification process, hydrologic conditions, water quality gradients, plants and so on, also affect the N_2_O emission when the conditions changed from flooding to non-flooding^38^,^39^, our previous studies including both mechanistic and process measurements have clearly shown that anammox bacteria do not produce N_2_O^4^,^17^.

These results bring into question the validity of present estimates of N_2_O emissions from China (419 Gg N yr^–1^) and globally (6 Tg N yr^–1^)^40^. These inaccurate calculations are based on an overrated amount of N_2_O emission from N-fertilizer application and inadequate land surface data^36^,^41^. The findings of omnipresent and significant anammox activity in Chinese inland waters and wetlands demonstrates that the distribution and ecological consequence of anammox in terrestrial ecosystems is of major importance, and warrants recalculations of the global N budget to which China is a considerable contributor. In other words, the N_2_O emissions in China, and most likely the rest of the world, must presently be overestimated. In future, the global nitrogen cycle model must include the key process parameters of anammox to improve the nitrogen balance.

## Methods

### Study site background

- China’s freshwater ecosystem is the fourth largest in the world. In this study, samples were collected from inland freshwater ecosystems for anammox sampling, including lakes, streams/rivers, lake riparian zones, river riparian zones, peatlands, swamps, reservoirs, paddy soil and constructed wetlands. The typical riparian zones in the Three Gorges Reservoir and Baiyangdian Lake, with oxic–anoxic interfaces, served as the preliminary investigation sites.
- The Three Gorges Reservoir located in south-central China and is 2,335 m long and 185 m deep (Supplementary Fig. 1). It is built on the Yangtze River, the world’s third longest river. The water level of the Three Gorges Reservoir can reach 175 m above sea level at total capacity to buffer floodwaters, mostly in June to August each year, while the lowest storage holds 145 m.
- Baiyangdian Lake, the largest natural freshwater lake in North China, is a lake group of about 140 lakes with a total area of about 366 km^2^ at water level 10.5 m above sea level (Supplementary Fig. 2). There are altogether 9,400 ha of reed fields with more than 3,700 ditches (approximately 24.8 km^2^) in Baiyangdian Lake, forming a characteristic reed-bed/ditch landscape.

Given the high heterogeneity of landscape, water quality, section of riparian zones, microbial biodiversity and activity in various types of inland waters and wetland ecosystems, a total of 256 samples were collected between June and December 2012, with different environmental backgrounds including nitrogenous compound content, plants, water quality, soil type, annual precipitation, and others, so as to make the research results representative for the whole country. Sample sites within China’s territory were from latitude 22 to 44 degrees north and longitude 80 to 120 degrees east.

## Methods summary

### Activity measurements

The occurrence, activity and contribution of anammox and denitrification to N_2_ production in sampling sites were measured in intact sediment/soil cores (0–50 cm) using the ^15^N-tracer technique^42^ at *in situ* temperature, combined with anoxic slurry assays^43^,^44^,^45^. Production rates of ^29^N_2_ and ^30^N_2_ in intact sediment/soil cores measured by Isotope Ratio Mass Spectrometers (Finnigan MAT 253, Germany), together with the determined *ra* values in the slurry incubations, were used to calculate total N_2_ production and anammox/denitrification rates (Supplementary Table 7).

### Molecular (q)PCR assay

A nested-PCR assay was conducted to detect anammox 16S rRNA genes according to established protocols^46^,^47^,^48^. The anammox sequences obtained in this study are available in NCBI under accession numbers GU083845-GU084118, JQ762016-JQ762251 and KC454442-KC454624. The abundances of anammox were determined by qPCR using the fluorescent dye SYBR-Green approach, targeting a subunit of the hydrazine synthase gene (*hzs*) which is specific for anammox^4^,^49^,^50^ with detailed information in Supplementary Table 8.

### N_2_O fluxes

N_2_O fluxes were determined in triplicate by a closed-chamber technique and gas chromatography. N_2_O flux was calculated from the linear change of its concentration in the chamber headspace. Coefficients of determination (*R*^2^) for linear regression of the concentration change over time were >0.90 for most data sets, with detailed information in ref. (51). 

## Additional Information

**How to cite this article**: Zhu, G. *et al.* Ubiquitous anaerobic ammonium oxidation in inland waters of China: an overlooked nitrous oxide mitigation process. *Sci. Rep.* **5**, 17306; doi: 10.1038/srep17306 (2015).

## Supplementary Material

Supplementary Information

## Figures and Tables

**Table 1:** Estimated budget of N loss by anammox in China inland waters and wetland ecosystem.

Type of Wetlands	Total area (km^2^)	Sample number	Anammox		Denitrification		Contribution of anammox to Total N loss (%)
			Average activity (μmol N m^−2^ h^−1^)	Total N loss (t yr^−1^)	Average activity (μmol N m^−2^ h^−1^)	Total N loss (t yr^−1^)	
	A	B	C	D	E	F	G
**N**_**2**_ **fluxes in various types of inland waters and wetland ecosystem in China**							
**Paddy field**	**1567000**	**55**	**5.8** ± 3.4	**(1.1** ± 0.7 **) × 10**^**6**^	**58.5** ± 34.1	**(1.1** ± 0.7 **) × 10**^7^	**10.14** ± 4.8
**River**	**31623**			**(1.1** ± 0.7 **) × 10**^5^		**(7.6** ± 5.0 **) × 10**^5^	**12.8** ± 8.0
* Open water*	*30763*	*21*	*28.3 *±* 20.5*	*(1.1* ± *0.7 ) *×* 10*^5^	*187.3* ± *106.9*	*(7.1* ± *5.0 ) *×* 10*^5^	*14.7* ± *7.9*
* Interface*	*860*	*17*	*37.6* ± *21.3*	*(4.0* ± *2.3 ) *×* 10*^*3*^	*480.0* ± *338.3*	*(5.1* ± *3.5 ) *×* 10*^4^	*8.4* ± *5.0*
**Swamp**	**81044**	**38**	**8.1** ± 6.6	**(8.1** ± 6.6 **) × 10**^4^	**77.7** ± 43.5	**(7.7** ± 4.3 **) × 10**^5^	**8.4** ± 3.8
**Lake**	**64395**			**(5.6** ± 3.5**) × 10**^5^		**(2.3** ± 1.2**) × 10**^**6**^	**19.3** ± 7.3
* Open water*	*64339*	*22*	*70.2* ± *44.4*	*(5.5* ± *3.5 ) *×* 10*^5^	*294.6* ± *153.1*	*(2.3* ± *1.2 ) *× *10*^*6*^	*19.2* ± *7.3*
* Interface*	*56*	*20*	*505.0* ± *222.8*	*(3.5* ± *1.5 ) *× *10*^*3*^	*686.0* ± *311.3*	*(4.7* ± *2.1 ) *× *10*^*3*^	*46.2* ± *15.9*
**Constructed wetland**	**12**	**25**	**333.6** ± 180.1	**(4.9** ± 2.7 **) × 10**^2^	**503.2** ± 172.0	**(7.4** ± 2.5 **) × 10**^2^	**38.0** ± 11.1
**Artificial pond**	**12487**			**(9.5** ± 2.2 **) × 10**^4^		**(1.7** ± 0.3 **) × 10**^5^	**36.3** ± 0.5
* Open water*	*12472*	*2*	*62.0* ± *14.3*	*(9.5* ± *2.2 ) *× *10*^4^	*108.6* ± *22.7*	*(1.7* ± *0.3 ) *× *10*^5^	*36.3* ± *0.5*
* Interface*	*15*	*8*	*15.2* ± *6.2*	*(2.8* ± *1.1 ) *×* 10*^*1*^	*89.0* ± *58.0*	*(1.6* ± *1.2 ) *× *10*^2^	*18.1* ± *8.1*
**Total N loss in China inland waters and wetland ecosystem**				**(2.0** ± 0.7 **) × 10**^**6**^		**(1.5** ± 0.7 **) × 10**^7^	**11.4** ± 5.0

NOTE: The detail information about the calculation of N loss was listed in Supplementary Table S5.
