# Supplementary material for: Ubiquitous anaerobic ammonium oxidation in inland waters of China: an overlooked nitrous oxide mitigation process
Source: Sci Rep. 2015 Nov 27;5:17306. doi: 10.1038/srep17306 (PMC4661425; doi:10.1038/srep17306)
Supplement: Supplementary Information [file srep17306-s1.pdf]

Ubiquitous anaerobic ammonium oxidation in inland waters of China: an overlooked nitrous oxide mitigation process

Guibing Zhu[1,4*], Shanyun Wang[1], Leiliu Zhou[1], Yu Wang[1], Siyan Zhao[1], Chao Xia[1], Weidong Wang[1], Rong Zhou[1], Chaoxu Wang[1], Mike S. M. Jetten[2], Mariet M. Hefting[3], Chengqing Yin[1], Jiuhui Qu[1]

1. Key Laboratory of Drinking Water Science and Technology, Research Center for Eco-Environmental Sciences, Chinese Academy of Sciences, Beijing 100085, China
2. Department of Microbiology, Radboud University Nijmegen, the Netherlands;
3. Ecology and Biodiversity Group, Department of Biology, Utrecht University, the Netherlands;
4. Department of Biogeochemistry, Max Planck Institute for Marine Microbiology, Bremen, Germany

* Corresponding author, E-mail: gbzhu@rcees.ac.cn

Supplementary Information

Summary

We provide here supplementary materials such as methodologies, complementary data, experimental analysis, supplementary figures and tables and detailed information of sampling sites. Detailed research method includes molecular (q)PCR, cloning and sequencing assay, measuring of anammox and denitrification rate with ^{15}N-tracer technique by intact core method and slurry incubation method, N_2O concentrations and fluxes measuring, and analytical procedures of environmental variables including physicochemical parameter and *in-situ* dissolved oxygen (DO). We also provided some supplementary figures and tables to illustrate the main text. The detailed information of sampling sites was listed in the last part.

Detailed research methods

DNA Extraction, PCR, Cloning and Sequencing Analysis

About 0.35 g freeze-dried sediment of each sample at each site was used for DNA extraction using a FastDNA SPIN Kit for Soil (Bio 101, USA) following the manufacturer's protocol with some modifications. After adding Sodium Phosphate Buffer and MT buffer to the lysing Matrix E tube, we homogenize it in the FastPrep@ Instrument for 45 seconds at a speed setting of 5.5 and centrifuge it at 14,000 ×g for 15 minutes. Additionally, we adjust the amount of DES to 75 μL. Specifically, we added 45 μL DES first, and after tapping the tube we injected the left 30 μL DES. The extracted DNA was checked on 1 % agarose gel and the concentration was determined with Nanodrop® ND -1000 ultraviolet-visible spectrophotometry (Thermo, USA). A nested-PCR assay was conducted to detect anammox 16S rRNA genes. PCR were performed in a C1000™ thermal cycler (BioRad, USA). The initial amplification was carried out using the PLA46f-630r primer combination with a thermal profile of 96 ℃ for 10 min, followed by 35 cycles of 60 s at 96 ℃, 1 min at 56 ℃, 1 min at 72 ℃. After the first step, a 500-times diluted (1 μl) PCR product was used as template for the second amplification with Amx368f-Amx820r primers using a thermal profile of 96 ℃ for 10 min, followed by 25 cycles of 30 s at 96 ℃, 1 min at 58 ℃, 1 min at 72 ℃. The PCR product was gel-purified and ligated into the pGEM-T Easy Vector (Promega, USA). The resulting ligation products were used to transform *Escherichia coli* JM109 competent cells following manufacturer instructions. In total, 20-62 clones were picked for each of the PCR product from one sampling site. PCR screens for the presence of inserts were performed using T7 and SP6 vector primers and the amplicons were analyzed with restriction endonuclease *Hha* I, *Hae* III and *Rsa* I (TAKARA, Dalian, China). Restriction digestion was carried out in a total volume of 20 μL including 5U restriction enzymes and 4 μL PCR products, and the system was incubated for 2 h at 37 ℃. Digested DNA fragments were analyzed by fragments separation on a 2 % (w/v) agarose gel and visualized with a GBOX/HR-E-M (Syngene, UK). Representative clones from each digestion

pattern were selected for sequencing using an ABI 3730XL automated sequencer (Applied Biosystems, USA). BLAST searches against the GenBank database verified that the PCR products were most closely related to aimed sequences. Plasmids were extracted with a GeneJet Plasmid Miniprep Kit (Fermentas, Lithuania). The plasmid DNA concentration was determined on a Nanodrops ND-1000 UV-Vis Spectrophotometer (NanoDrop Technologies, Wilmington, DE, USA) for calculation of *hzs* gene copy number. The standards and the DNA samples were performed on the same plate. All the sequences and their relatives obtained from the NCBI BLAST were aligned by using the Clustal X1.83 program (Thompson *et al.*, 1997). The anammox bacterial sequences sharing 97 % nucleotide similarity were grouped into the same operational taxonomic unit (OTU) using DOTUR software by employing the furthest neighbor approach (Schloss and Handelsman 2005). The biodiversity indicator (Shannon and Chao 1) were also calculated with DOTUR software. Phylogenetic trees were constructed by neighbor-joining (NJ) with the Jukes–Cantor correction using the MEGA 4 package (Tamura *et al.*, 2005). The neighbor-joining phylogenetic tree showing the phylogenetic affiliations of anammox 16S rRNA gene sequences from various inland waters with bootstrap values of 1,000 replicates. The detailed information of PCR amplification protocols and cloning for anammox are described in our previous work (Zhu *et al.*, 2013).

Quantitative Real-Time PCR

The abundances of anammox were determined by qPCR using the fluorescent dye SYBR-Green approach, targeting a subunit of the hydrazine synthase gene (*hzs*) which is specific for anammox. SYBR Green I based real-time PCR assays were carried out in a mixture of 20 µL, containing 10 µL SYBR® *Premix Ex Taq*™ (TAKARA, Dalian, China), 4 pmol of each primer and 2 µL of 10-fold diluted DNA template. Amplification and detection were carried out with an ABI Prism 7300 Sequence Detection System (Applied Biosystems, USA) with the primer sequences and Thermal profiles compiled in supplementary Table 8. Three no-template controls (NTCs) were run

for each quantitative PCR assay. Tenfold serial diluted plasmid DNA with known copy number was subjected to real-time PCR in triplicate to generate an external standard curve. Melting curves were generated after each assay to check the specificity of amplification. PCR efficiencies were 90-103 % (average 92 %) for anammox bacterial hydrazine synthase gene (*hzs*B) and archaeal & bacterial *amo*A genes**.** Only the results with correlation coefficient above 0.98 were employed.

In the real-time PCR quantitative assays targeting the *hzs*B gene, the detection limit of environmental samples was determined by a diluting method. Four samples with the lowest abundance were chosen and diluted for 10 times, 20 times, 60 times, 100 times, 500 times. With the identical PCR procedure, the lowest anammox abundance was observed with the undiluted sample of CZ29-4 at 8.877 copies/μl (supplementary Figure 7) which was thus assumed to be the detection limit in this environmental investigation.

Measuring anammox and denitrification rates with ^{15}N-tracer technique

The anammox and denitrification rates were obtained in intact incubations with ^{15}N-tracer technique combined with the measuring of anammox and denitrification potentials in slurry incubations (Trimmer *et al.*, 2006). Intact sediment/soil cores were collected from all the sampling sites. The 10 cm long Plexiglas core tubes with an i.d. of 5.0 cm were used for sediment/soil sampling and incubation. Cores were capped and stored at 4 ℃ then returned to the laboratory for pre-incubation by being placed in an open tank filled with air-saturated *in situ* water and maintained at *in situ* temperatures. Small Teflon coated magnets were placed 5 cm above the sediment/soil surface and rotated by an external magnet (≈ 60 rpm) to ensure a homogenous mixing of the water column. After 12 h pre-incubation, a stock solution of $^{15}NO_3^-$ (99.29%) was added to the water in the open incubation tank to achieve a final concentration of about 100 μM, and a syringe was used to exchange the water in each sediment/soil core with $^{15}NO_3^-$ rich water from the reservoir in order to obtain a uniform mixing of the added isotope in all cores. Gastight lids were then secured on all cores and incubation then started. Three amended cores were sacrificed at time of 0, 3, 6, 12 and 24

h by opening the lids, gently stirring the sediment/soil and the ambient water, and collecting 12 mL of slurry into a gastight vial (Exetainer, Labco, UK, 12 mL) containing 200 μL of 7 M $ZnCl_2$, for N_2 analysis. Samples in vials were then capped without headspace.

At the same time, anoxic slurry assays with ^{15}N-tracer technique were also conducted according to reference (Risgaard-Petersen *et al.*, 2004). The homogenized sediment/soil samples with known weight (2 mL, about 3-3.5 g) and density were transferred to the 12-mL gastight vials (Exetainer, Labco, UK) together with N_2-purged media water at *in situ* temperature. The resulting slurries were then pre-incubated for 24 h to remove residual NO_x^- in sediments/soils and incubation media. Subsequently, 100 μL of N_2-purged stock solution of each isotopic mixture, i.e. (1) $^{15}NH_4^+$ (^{15}N at.%: 99.60), (2) $^{15}NH_4^+$ + $^{14}NO_3^-$ and (3) $^{15}NO_3^-$ (^{15}N at.%: 99.29) was added to each parallel slurry samples resulting in a concentration of about 100 μM N. Incubation of three of the slurries was stopped at 0, 3, 6, 12 and 24 h by adding 200 μL of a 7 M $ZnCl_2$ solution. In case of the slurries amended with $^{15}NH_4^+$ only, no significant accumulation of ^{15}N-labeled gas ($^{29}N_2$ and/or $^{30}N_2$) could be observed in any sample, indicating that all ambient $^{14}NO_x^-$ had been consumed during the 24-h pre-incubations. When both $^{15}NH_4^+$ and $^{14}NO_3^-$ were added, $^{29}N_2$ accumulated in every sediment sample and interface soil without any accumulation of $^{30}N_2$. This pattern was reproducible and the results showed that the anammox process was detectable in the sediments and interface soils. Slurries amended solely with $^{15}NO_3^-$ were analyzed by measuring the ^{15}N-labled N_2 production for anammox and denitrification potentials.

For N_2 analysis both for intact and slurry incubations, a 2-mL clarified water sample from gastight vials containing ^{15}N treated samples was introduced into another gastight vial flushed with analytical grade He. The vials were then shaken vigorously, inverted, and stored upright at 22 ℃ to allow N_2 to equilibrate between the water phase and headspace. Headspace gas samples were then analyzed for $^{28}N_2$, $^{29}N_2$ and $^{30}N_2$ content measured by Isotope Ratio Mass Spectrometers (Finnigan MAT 253, Germany). The measurements were accomplished in the key laboratory of Tibetan environment changes and land surface processes, Institute of Tibetan Plateau Research, Chinese

Academy of Sciences.

The rates and potential contributions to N_2 formation of either anammox or denitrification were calculated from the produced $^{29}N_2$ and $^{30}N_2$. Firstly, to obtain the contribution of anammox to total N_2 production (*ra*), potential rates of anammox and denitrification in the slurry incubations were calculated using the equations described by Thamdrup and Dalsgaard (Equation 1# and 2# in supplementary Table 7). Then we quantified anammox and denitrification in intact cores using the method, in which production rates of $^{29}N_2$ and $^{30}N_2$, together with the determined r_{14} value (Equation 3# in Table S7) were then used to calculate total N_2 production and anammox/denitrification rates (Equation 4#, 5# and 6# in supplementary Table 7).

N_2O concentrations measuring and fluxes

The closed-chamber technique was applied to measure nitrous oxide, with triplicate chambers at the each time. The stainless steel chambers consisted of two parts: pedestal and upper chamber. The pedestal is 25 cm high with an internal diameter of 40 cm. The lower rim was sharpened to be driven into soil, and the upper rim had a 2 cm by 2 cm gutter around the outside that could be filled with water to make an airtight seal within the upper chambers. The upper chamber (h = 45 cm) was equipped with two battery driven brushless fans, for mixing of the chamber headspace, and one temperature probe.

N_2O concentrations were measured shortly after sampling by gas chromatography (Agilent 4890D) with an electron capture detector (ECD). The temperature of the ECD was 330 ℃ and the oven was 55 ℃. The precision of the N_2O analyses was ±2.8 %, based on replicate analysis of standard gas. When temperature was 25 ℃ and air pressure was 1000 hPa, the minimum detectable fluxes were 28 mg m^{-2} h^{-1}, 14 mg m^{-2} h^{-1} and 9 mg m^{-2} h^{-1} in 5 min, 10 min and 15 min gas sampling intervals, respectively. A standard gas was analyzed after every 6 samples. N_2O flux was calculated from linear change of its concentration in chamber headspace as a function of time, base area, chamber volume, and molar volume of N_2O at chamber headspace air temperature. Values of

the coefficient of determination (R^2) for linear regression of the concentration change over time were greater than 0.90 for most data sets.

Statistical analysis

Statistical analyses were conducted using PASW Statistics 18.0 software (Predictive Analytics Software Statistics). The Kruskal-Wallis test and Mann-Whitney U test were used respectively for the comparison of three and two data groups. Correlations between variables were computed by Spearman correlation analysis. The level of significance in this study was $\alpha = 0.05$. Graphing was achieved using Origin 8.0 software. The variability of determined anammox bacterial abundance, rates and N_2O flux were expressed as interquartile range, the most commonly-used resistant measure of spread, which was defined as the 75th percentile minus the 25th percentile.

Analytical procedures of environmental variables

All the analyses of water quality parameters were performed according to the Standard Methods (APHA). The measurements of DO, pH and the temperature of water were conducted using a multi 340i device (WTW, Germany), equipped with CellOx 325 and pH-Electrode SenTix 4 probes, respectively. The environmental physicochemical variables of sediments and soils, including pH, NH_4^+-N, NO_x^--N, total nitrogen, total phosphorus, total organic material, total carbon, were investigated according to ref (Bao 2000). The dissolved oxygen concentration in surface sediments was measured *in situ* using an OXY Meter S/N 4164 with stainless electrode sensor (Unisense, Aarhus, Denmark), according to ref (Gundersen *et al.*, 1998). Triplicates were run for QA/QC.

Supplementary figures and tables

Figure 1 Geographical and detailed information of the sampling sites in the Water Level Fluctuation Zone of Three Gorges Reservoir. From section *a* to *d* the figures show the geographical location of the sampling site in China and Three Gorges Reservoir (***a***), the

Fluctuations of water level, flooding duration of sampling sites and sampling time during 2013.5 to 2014.8 (***b***), the landscape from far (***c***) and close viewing (***d***) and Dr. Yu Wang when sampling (***e***). The map were come from web of "Data Sharing Infrastructure of Earth System Science" http://www.geodata.cn. The photograph was taken by Guibing Zhu with the permission of Yu Wang.

Figure 2 Geographical and detailed information of Baiyangdian Lake and sampling sites. From section *a* to *d* the figures show the geographical location of Baiyangdian Lake in China drawn with software ArcGIS (***a***), the Fluctuations of water level, flooding duration of sampling sites and sampling time drawn with software EXCEL (***b***), the landscape taken by author Weidong Wang(***c***) and vertical section drawn by software CAD(***d***) of reed-bed/ditch systems in Baiyangdian Lake, and the sampling picture of Dr. Shanyun Wang and Lei Ye taken by Guibing Zhu with the permission of Shanyun Wang and Lei Ye (***e***). The map were come from web of "Data Sharing Infrastructure of Earth System Science" http://www.geodata.cn. All of the maps used in the manuscript are free.

Figure 3 Vertical distribution of key physicochemical parameters in Jiaxing paddy soil (0-100 cm, ***a***), North Canal sediments (0-50 cm, ***b***) and Baiyangdian Lake sediments (0-20 cm, ***c***)

Figure 4 Graphs showed how we got the rates of anammox and denitrification under different substrate conditions. Three treatments of a sample with ^{15}N labeled on ammonium and nitrate separately were conducted to verify the exhaustion of nitrate or nitrite (A-*a*), confirm the occurrence of anammox (A-*b*), and calculate the anammox and denitrification rates (A-*c*). When the substrates were **insufficient**, the N_2 production by anammox and denitrification would stagnate (B), and then the rates of anammox and denitrification were calculated using data before the stagnation (C). When the substrates were **sufficient**, the rates were calculated directly from the slopes between N_2 production and time (D).

Figure 5 Biogeographical distribution of anammox bacterial abundance and specific cellular rate in

China Inland Waters. The map were come from web of “Data Sharing Infrastructure of Earth System Science” http://www.geodata.cn. All of the maps used in the manuscript are free. With the map we use the EXCEL software to draw the column or pie at the same bar scale and paste them on the sampling site in the map to create the figure.

Figure 6 Information of quantitative PCR indicating plots of the standard curve (***a***), the slope was -3.42, and R^2 was 0.99198), the amplification plots of standards samples, negative control and environmental sample of detection limit (***b***),the melting curves of the standards samples (***c***) and the melting curve of detection limit sample CZ29-4 (***d***).

Table 1 The biogeographic background of samples in China inland waters and wetland systems

Table 2 The physicochemical parameters of sampled sediments and soils in various inland waters and wetland systems

Table 3 Spearman correlation matrix between anammox rates and the physicochemical parameters

Table 4 Spearman correlation matrix between anammox rates and some biogeographic parameters

Table 5 Equations used for the estimated budget of N loss by anammox in China inland waters and wetland ecosystem[a]

Table 6 Spearman correlation between anammox abundance and N_2O flux

Table 7 Equations used for the calculation of anammox and denitrification rates

Table 8 Primers used in this study and correspondence thermal profiles

a

b

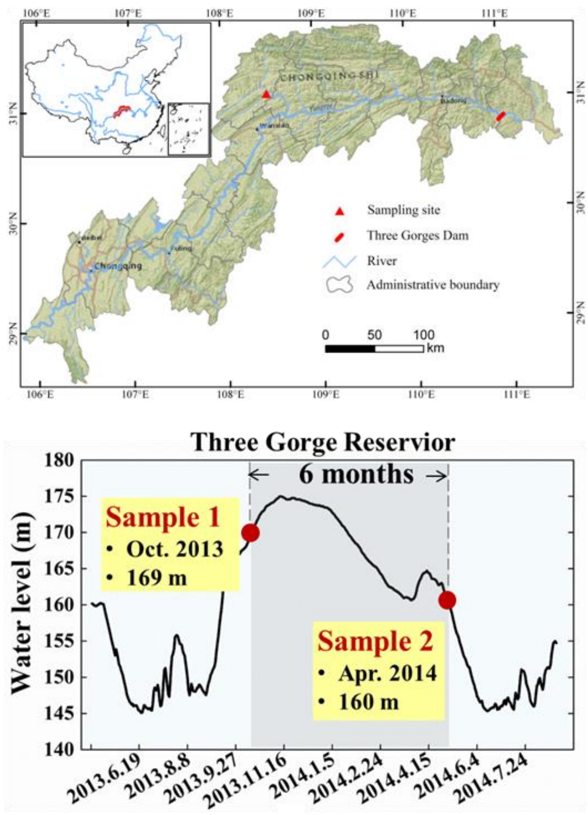

c

d

e

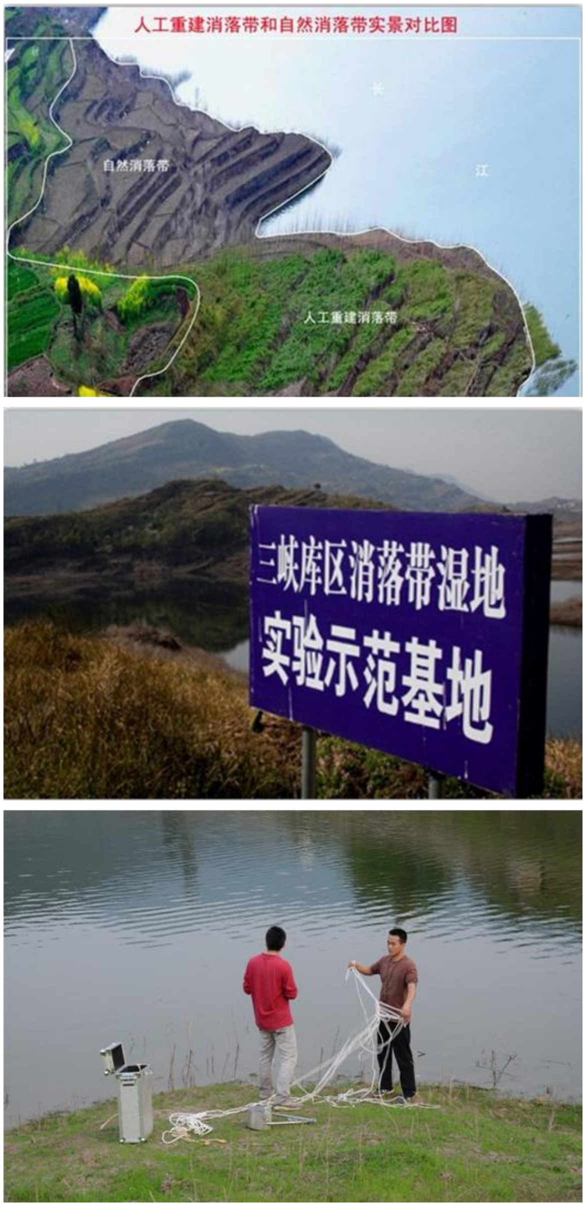

Figure 1 Geographical and detailed information of the sampling sites in the Water Level Fluctuation Zone of Three Gorges Reservoir. From section *a* to *d* the figures show the geographical location of the sampling site in China and Three Gorges Reservoir (***a***), the Fluctuations of water level, flooding duration of sampling sites and sampling time during 2013.5 to 2014.8 (***b***), the landscape from far (***c***) and close viewing (***d***) and Dr. Yu Wang when sampling (***e***). The map were come from web of "Data Sharing Infrastructure of Earth System Science" http://www.geodata.cn. The photograph was taken by Guibing Zhu with the permission of Yu Wang.

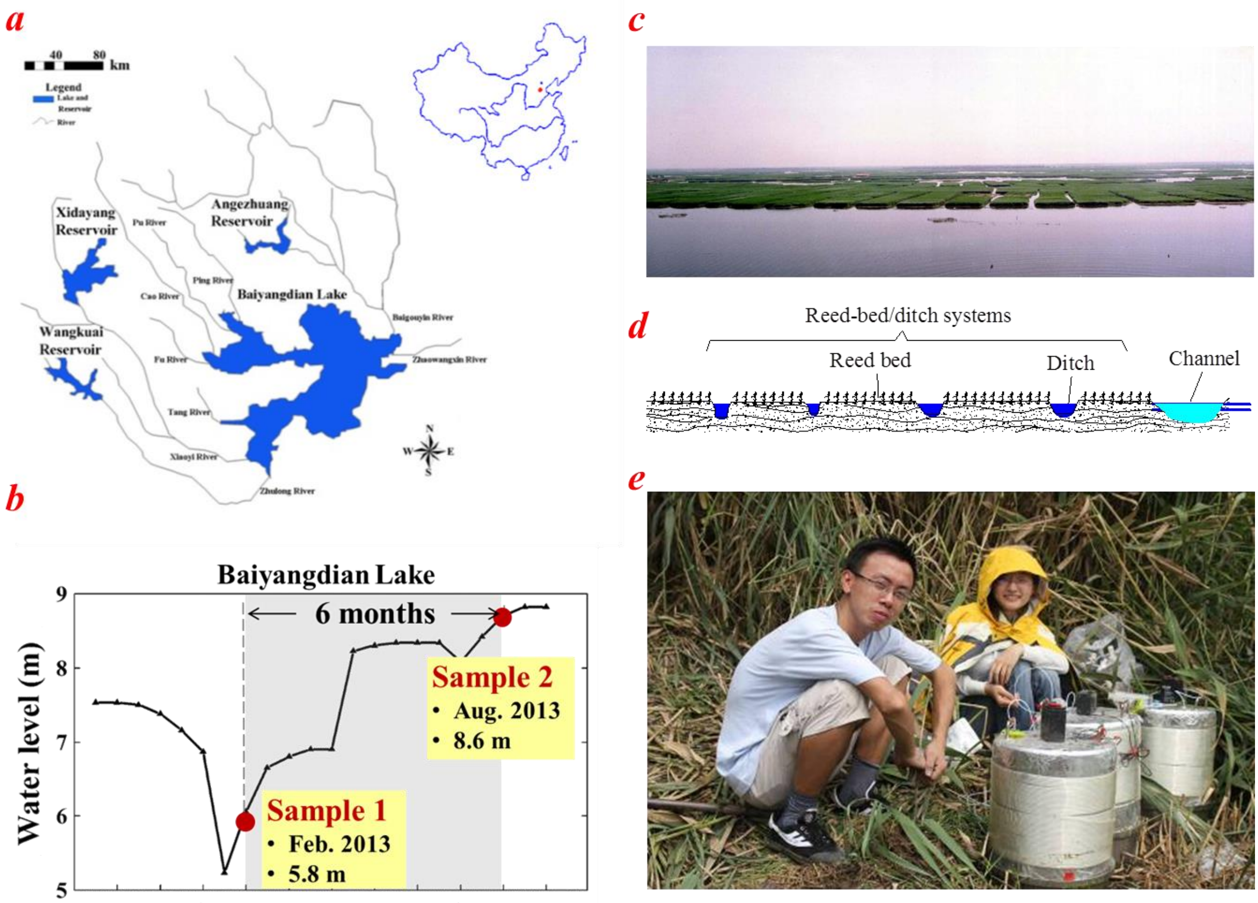

Figure 2 Geographical and detailed information of Baiyangdian Lake and sampling sites. From section *a* to *d* the figures show the geographical location of Baiyangdian Lake in China drawn with software ArcGIS (***a***), the Fluctuations of water level, flooding duration of sampling sites and sampling time drawn with software EXCEL (***b***), the landscape taken by author Weidong Wang(***c***) and vertical section drawn by software CAD(***d***) of reed-bed/ditch systems in Baiyangdian Lake, and the sampling picture of Dr. Shanyun Wang and Lei Ye taken by Guibing Zhu with the permission of Shanyun Wang and Lei Ye (***e***). The map were come from web of "Data Sharing Infrastructure of Earth System Science" http://www.geodata.cn. All of the maps used in the manuscript are free.

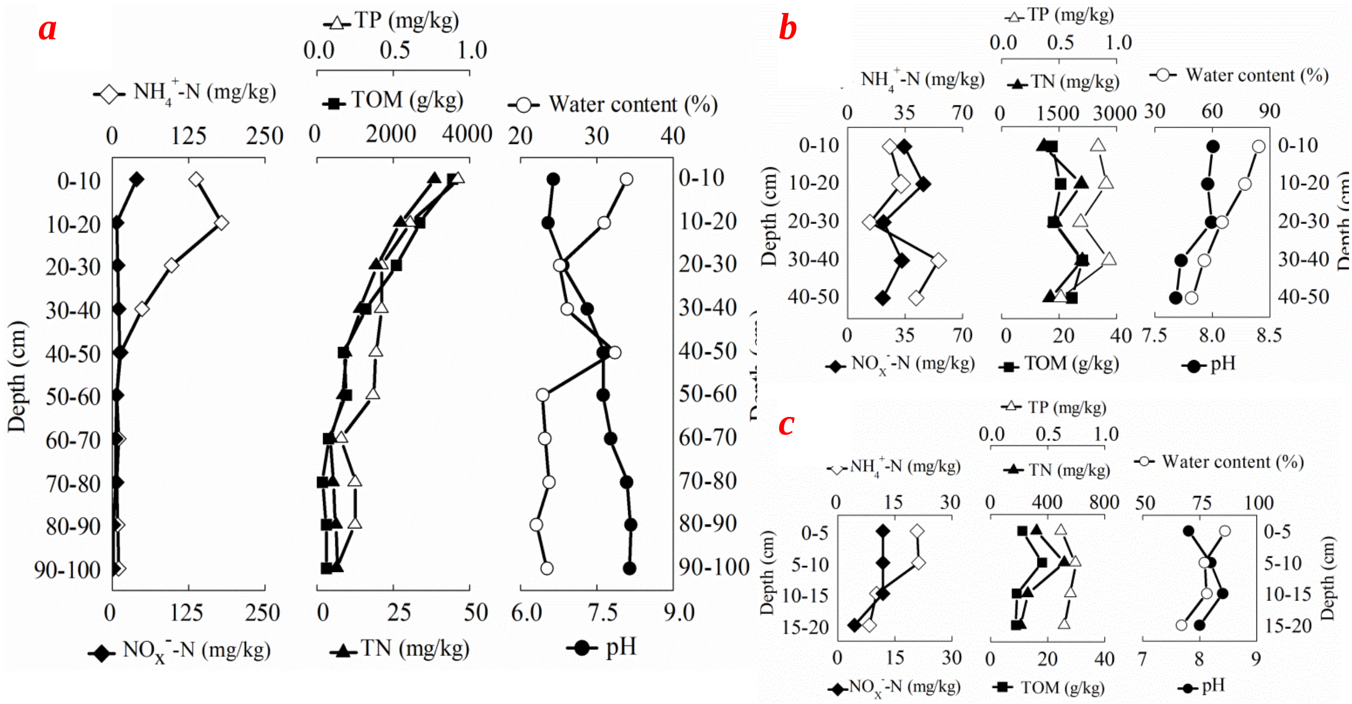

Figure 3 Vertical distribution of key physicochemical parameters in Jiaxing paddy soil (0-100 cm, *a*), North Canal sediments (0-50 cm, *b*) and Baiyangdian Lake sediments (0-20 cm, *c*)

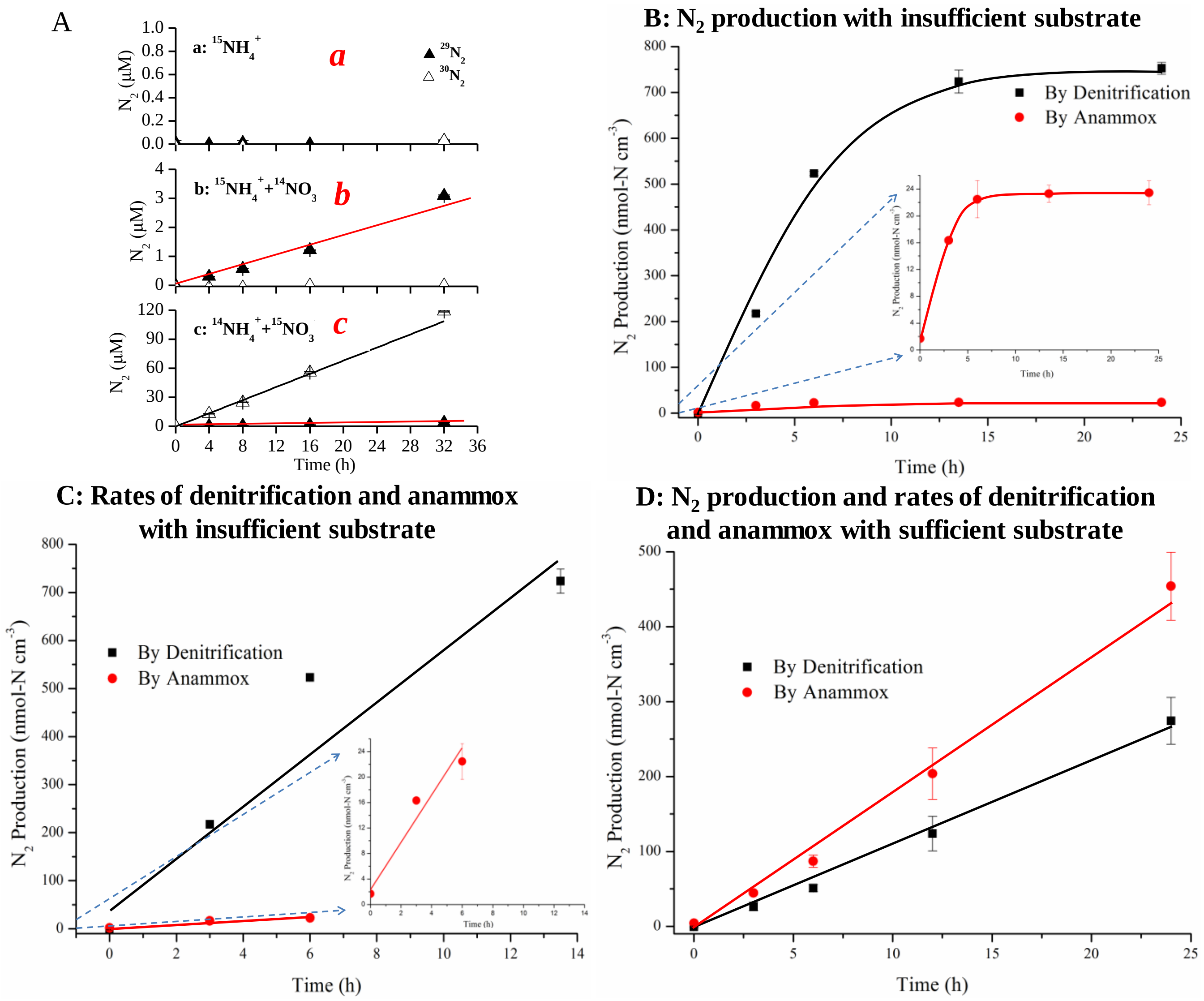

Figure 4 Graphs showed how we got the rates of anammox and denitrification under different substrate conditions. Three treatments of a sample with ^{15}N labeled on ammonium and nitrate separately were conducted to verify the exhaustion of nitrate or nitrite (A-*a*), confirm the occurrence of anammox (A-*b*), and calculate the anammox and denitrification rates (A-*c*). When the substrates were **insufficient**, the N_2 production by anammox and denitrification would stagnate (B), and then the rates of anammox and denitrification were calculated using data before the stagnation (C). When the substrates were **sufficient**, the rates were calculated directly from the slopes between N_2 production and time (D).

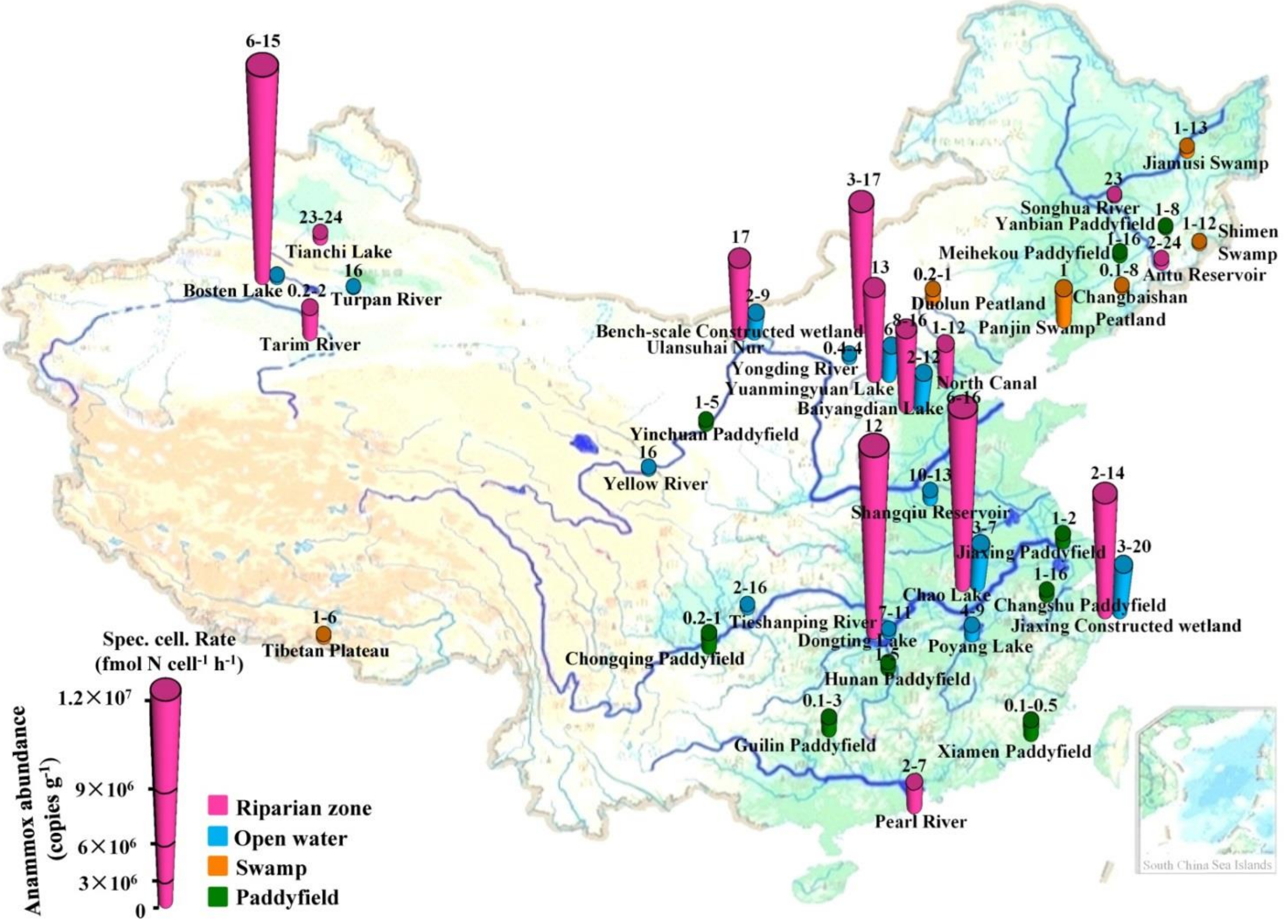

Figure 5 Biogeographical distribution of anammox bacterial abundance and specific cellular rate in China Inland Waters. The map were come from web of "Data Sharing Infrastructure of Earth System Science" http://www.geodata.cn. All of the maps used in the manuscript are free. With the map we use the EXCEL software to draw the column or pie at the same bar scale and paste them on the sampling site in the map to create the figure.

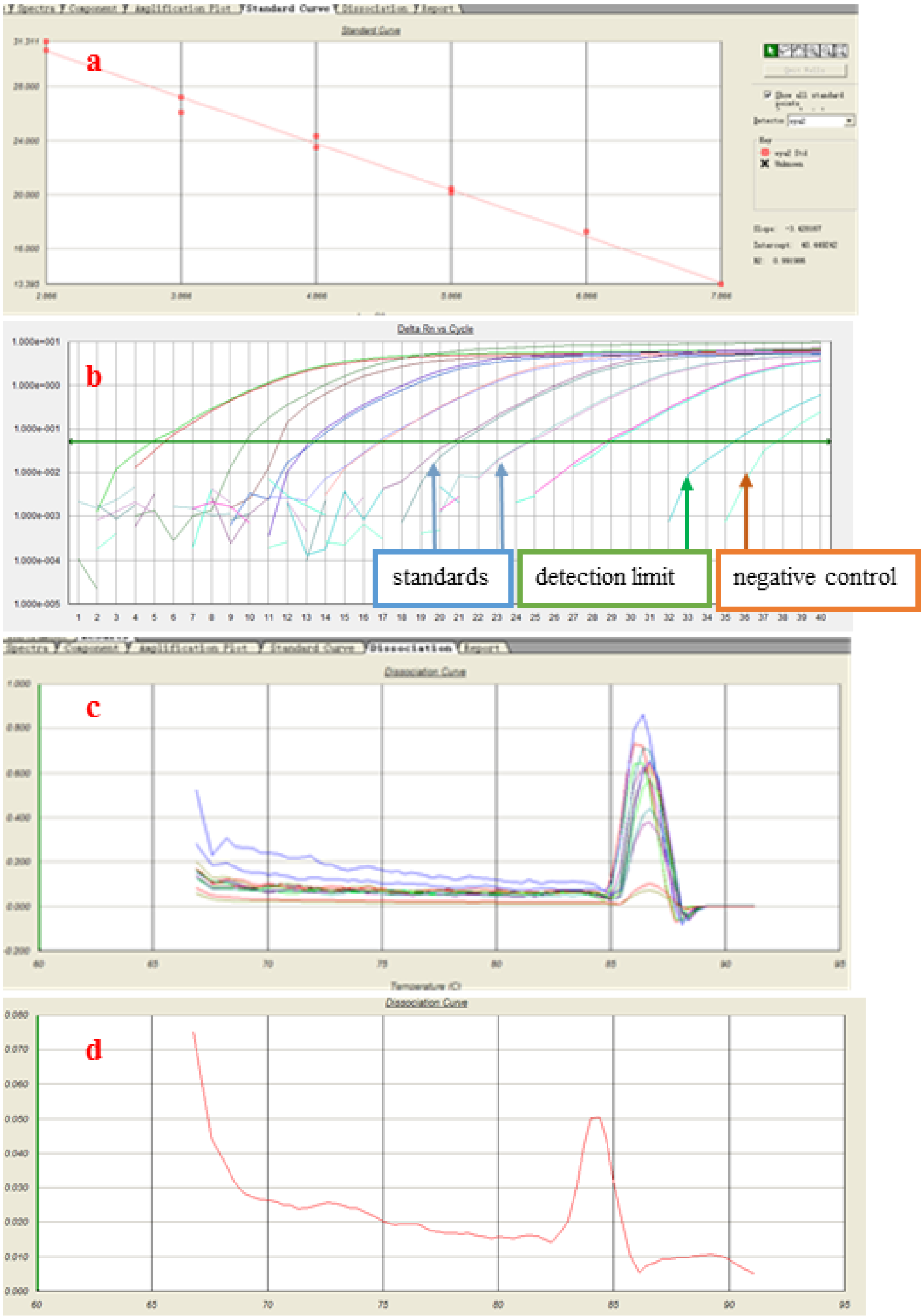

Figure 6 Information of quantitative PCR indicating plots of the standard curve (***a***, the slope was -3.42, and R^2 was 0.99198), the amplification plots of standards samples, negative control and environmental sample of detection limit (***b***),the melting curves of the standards samples (***c***) and the melting curve of detection limit sample CZ29-4 (***d***)

Table 1 The biogeographic background of sampled inland water bodies and wetland systems

No.	Inland waters	Coordinates	Wetlands background		Number of samples	Geography climatic factors						
			Type	Sampling environments and backgrounds		Elevation (m)	Precipitation (mm)	Average annual temperature (°C)	Diurnal temperature range (°C)	Sunshine duration (h)	Climate classification	Soil classification
**01**	Tarim River	41°03′-41°04′N, 86°06′-86°07′E	Riparian	The longest endorheic river in China and 5th in the world	4	890.00	41.65	11.03	15.23	3147.23	Warm temperate continental desert climate	Gray desert soil
**02**	Bosten	41°49′-41°54′N, 86°43′-86°57′E	Lake riparian	The largest endorheic freshwater lake in China	4	1050.00	64.70	7.90	15.00	3109	Warm temperate continental desert climate	Gray desert soil
**03**	Tianchi	43°54′N, 88°08′E	Lake riparian	Lake with high elevation and low temperature	2	1922.00	594.38	2.23	10.45	2549.18	Warm temperate continental desert climate	Sierozem Brown calcareous
**04**	Turpan River	42°28′N, 89°12′E	Lake	The lowest site (-154 m) in China; 2nd of the world	3	176.00<br>289.00<br>223.00	15.82	14.52	13.37	2941.52	Warm temperate continental desert climate	Gray desert soil
**05**	Tibetan Plateau	28°53′20″-28°55′05″N 90°20′10″-90°22′30″E	Swamp	The highest altitude is over 5200 m	8	4600.00	376.00	8.10	14.24	2929.7	Temperate steppe climate	Subalpine steppe soil
**06**	Yellow River	36°05′N, 103°46′E	River	The second largest river in China	2	1527.00	319.40	9.58	12.52	2503.14	Warm temperate continental grassland climate	Chernozem chestnut soil Dark loessial soil
**07**	Yinchuan	38°20′06″-38°21′30″N, 106°21′20″-106°23′30″E	Paddyfield	Irrigated with the Yellow river water	8	1110.00	193.64	9.00	12.79	2906.23	Mid-temperate continental desert steppe climate	Chernozem chestnut soil Dark loessial soil
**08**	Wuliangsuhai	40°53′-40°55′N, 108°49′-108°52′E	Lake	The largest wetland on the same latitude in the world	4	1022.00	207.18	6.50	13.49	3202	Mid-temperate continental steppe climate	Chernozem chestnut soil Dark loessial soil
**09**	Tieshanping	29°36′00″-29°36′30″N 106°40′20″-106°41′10″E	River	Catchment area in the watershed that is polluted by acid rain	7	532.00	1246.35	16.69	7.35	1303.7	Mid-subtropical monsoon evergreen broad-leaved forest climate	Red soil Yellow soil
**10**	Jiamusi	46°06′43″-46°09′07″N 130°14′31″-130°27′14″E	Swamp	The biggest swamp wetland in China	8	130.00	540.18	3.40	11.55	2464.45	Mid-temperate monsoon coniferous and broad-leaved mixed forests	Dark brown soil Black soil

											climate	
**11**	Songhuajiang	44°04′-46°40′N, 125°42′-130°10′E	River riparian	The largest tributary of Heilongjiang River	3	120.00	532.49	4.22	11.31	2511.07	Mid-temperate monsoon forest steppe climate	Dark brown soil Black soil
**12**	Yanbian	42°06′59″-42°07′19″N 128°54′00″-128°54′15″E	Paddyfield	Rice planting once a year; samples were taken before rice planting	8	385.00	594.70	3.60	12.55	2318.87	Mid-temperate monsoon coniferous and broad-leaved mixed forests climate	Dark brown soil Black soil
**13**	Shimen	43°02′05″-42°03′10″N 128°59′03″-129°00′45″E	Swamp	Located in the tributary of Buerhatong River	8	315.00	594.70	3.60	12.55	2318.87	Mid-temperate monsoon coniferous and broad-leaved mixed forests climate	Dark brown soil Black soil
**14**	Antu	43°03′55″-43°05′45″N 128°50′05″-128°52′10″E	Reservoir	Used as the source of drinking water	8	380.00	594.70	3.60	12.55	2318.87	Mid-temperate monsoon coniferous and broad-leaved mixed forests climate	Dark brown soil Black soil
**15**	Meihekou	42°26′40″-42°27′27″N 125°37′15″-125°39′04″E	Paddyfield	Rice planting once a year; samples were taken when planting rice	8	330.00	699.55	5.19	11.82	2468.63	Mid-temperate monsoon coniferous and broad-leaved mixed forests climate	Dark brown soil Black soil
**16**	Changbaishan	42°00′35″-42°02′40″N 127°25′40″-127°28′50″E	Peatland	Produced from the decomposition and deposition of sedges and other hydrophyte in marshland for thousands of years	8	860.00	518.33	5.32	12.55	2318.87	Mid-temperate monsoon coniferous and broad-leaved mixed forests climate	Dark brown soil Black soil
**17**	Duolun	42°13′N, 116°29′E	Peatland	Scattered sedge peatland in grassland	3	1235.00	379.52	2.19	13.56	3018.22	Mid-temperate monsoon forest steppe climate	Chernozem chestnut soil Dark loessial soil
**18**	Panjin	40°39′-41°27′N, 121 °25-122°31′E	Swamp	One of the wetlands preserved the best in the world	3	0.00	645.00	8.30	8.92	2726	Warm temperate monsoon deciduous broad-leaved mixed forest climate	Brown soil Cinnamon soil
**19**	Bench-scale wetland	40 °00'31"N, 116 °20'18"E	Constructed wetlands	Used to verify the findings in natural wetlands: horizontal flow<br>Intermittent flow	5<br>5	53.00	628.90	12.50	10.80	2662	Warm temperate monsoon deciduous broad-leaved mixed forest climate	Brown soil Cinnamon soil
**20**	Guilin	24°15′-26 °23′N, 109 °36′-111 °29′E	Paddyfield	Two rice-growing seasons	7	150	1949.5	18.9	6.3	1670	Mid-subtropical humid monsoon	Red soil

											climate	
**21**	Chongqing	28°10′-32°13′N, 105°11′-110°11′E	Paddyfield	rain-fed paddy fields with rice-fallow rotation system	3	400	1225	18	6.6	1100	Subtropical humid monsoon climate	Red soil
**22**	Xiamen	24°26'46"N, 118°04'04"E	Paddyfield	One rice-growing seasons	3	201	1200	20.6	6.7	2233.6	Subtropical oceanic climate	Lateritic red earth
**23**	Jiaxing	30°21′-31°02′N, 120°18′-121°16′E	Paddyfield	A paddy field with a high load of slurry manure as fertilizer	3	3.7	1168.6	15.9	7.78	2017	Subtropical monsoon climate	Red soil
**24**	Yuanmingyuan	40°00′N, 116°18′E	Lake<br>Lake riparian	Artificial lake feeding with reclaimed water	1<br>1	46.00<br>43.00	628.90	12.50	10.80	2662	Warm temperate monsoon deciduous broad-leaved mixed forest climate	Brown soil Cinnamon soil
**25**	Yongding	40°02′-40°03′N, 115°48′-115°50′E	River	The largest river in Beijing	3	321.00	528.70	10.20	10.80	2470	Warm temperate monsoon deciduous broad-leaved mixed forest climate	Brown soil Cinnamon soil
**26**	North Canal	40°04′N, 116°31′E	River riparian	Sediment from subsurface -10 - -20 cm: Summer<br>Winter	5<br>5	33.00	625.00	11.50	11.40	2750	Warm temperate monsoon deciduous broad-leaved mixed forest climate	Brown soil Cinnamon soil
**27**	Baiyangdian	38°54′-38°55′N, 115°56′-115°59′E	Lake<br>Lake riparian	The largest lake of North China	5<br>5	7.00	552.27	12.10	10.76	2638.3	Warm temperate monsoon deciduous broad-leaved mixed forest climate	Brown soil Cinnamon soil
**28**	Shangqiu	34°37'49"N, 115°57'54"E	Reservoir	Now has been subjected to non-point source pollution	2	49.00	707.59	14.16	10.42	2183.68	Warm temperate monsoon deciduous broad-leaved mixed forest climate	Brown soil Cinnamon soil
**29**	Changshu	31°28′41″-31°33′00″N 120°38′06″-120°41′10″E	Paddyfield	Irrigated by polluted river water	7	2.00	1054.00	15.40	7.78	2130.2	North Subtropical monsoon deciduous evergreen broad-leaved mixed forest climate	Yellow brown soil Yellow cinnamon soil
**30**	Jiaxing	30°46′-30°47′N, 120°42′-120°43′E	Constructed wetlands	The largest constructed wetlands in China: Interface Summer<br>Interface Winter<br>Waterward	5<br>5<br>5	7.00	1168.60	15.90	7.78	2017	North Subtropical monsoon deciduous evergreen broad-leaved mixed forest climate	Red soil Yellow soil
**31**	Chaohu	31°33′-31°41′N, 117°24′-117°47′E	Lake<br>Lake riparian	The fifth largest freshwater lake in China	7<br>7	5.00	1053.84	16.20	7.72	1977.85	North Subtropical monsoon deciduous evergreen broad-leaved mixed forest climate	Yellow brown soil Yellow cinnamon soil

**32**	Poyang	29°24′-29°26′N, 116°01′-116°02′E	Lake	The largest freshwater lake in China	3	9.00	2052.00	18.10	9.06	1736.74	Mid-subtropical monsoon evergreen broad-leaved mixed forest climate	Red soil Yellow soil
**33**	Dongting	29°20′-29°22′N, 113°05′-113°06′E	Lake riparian	The second largest freshwater lake in China	3	25.00	1325.91	17.24	6.40	1741.45	North Subtropical monsoon deciduous evergreen broad-leaved mixed forest climate	Red soil Yellow soil
**34**	Hunan	29°21′20″-29°22′00″N 113°11′35″-113°12′40″ E	Paddyfield	Plant rice in Summer, wheat in Winter	8	50.00	1325.91	17.24	6.40	1741.45	North Subtropical monsoon deciduous evergreen broad-leaved mixed forest climate	Red soil Yellow soil
**35**	Pearl River	23°08′-23 °09′N, 113°10′-113°11’E	Estuary	The third largest river in China: Summer<br>Winter	3<br>3	30.80<br>13.50<br>12.50	1743.32	22.08	7.53	1721.97	South subtropical monsoon rainforest climate	Lateritic red soil

Table 2 The physicochemical parameters of sampled sediments and soils in various inland water bodies and wetland systems

	Wetlands	pH		NH_4^+ (mg kg^{-1})		NO_x^- (mg kg^{-1})		TN (g kg^{-1})		TP (mg kg^{-1})		TOM (g kg^{-1})		TC (g kg^{-1})		TS (g kg^{-1})		DO in surface sediment (mg L^{-1})	
		Riparian sediment	Waterward sediment	Riparian sediment	Waterward sediment	Riparian sediment	Waterward sediment	Riparian sediment	Waterward sediment	Riparian sediment	Waterward sediment	Riparian sediment	Waterward sediment	Riparian sediment	Waterward sediment	Riparian sediment	Waterward sediment	Riparian sediment	Waterward sediment
**01**	Tarim River	8.45		16.73		4.29		0.38		1.26		5.02		32.89		0.38		0.30	
		8.61		20.47		5.12		0.30		1.24		2.48		29.42		0.31		0.27	
		8.68		14.10		2.64		0.31		1.26		3.78		30.37		0.31		0.28	
		8.91		29.63		5.02		0.15		1.04		0.69		22.85		0.33		0.34	
**02**	Bosten Lake	7.95	8.57	86.62	90.54	23.99	6.81	2.31	0.75	1.23	0.66	48.15	13.07	54.33	41.63	5.38	1.96	0.24	0.68
		8.16		76.21		22.58		3.16		1.29		78.14		61.85		9.78		0.18	
		8.15		109.93		17.82		1.40		1.04		29.37		63.33		1.65		0.21	
**03**	Tianchi Lake		7.66		0.81		0.40		1.52		0.5		25.52		34.36		0.73		0.25
			9.27		0.98		0.48		1.84		0.61		30.88		37.38		0.89		0.29
**04**	Turpan River		8.54		6.48		0.95		0.73		1.33		2.06		12.07		0.69		0.61
			7.89		7.97		1.16		0.90		1.63		2.54		14.85		0.84		0.64
			8.21		14.34		2.10		1.61		2.94		4.57		26.73		1.52		0.55
**05**	Tibetan Plateau Swamp		7.38		8.08		0.10		0.12		0.49		210.98		331.62		0.12		0.45
			7.45		5.57		1.04		0.12		0.50		211.49		332.00		0.12		0.15
			7.66		11.70		1.98		0.06		0.41		320.27		559.03		0.13		0.24
			7.38		8.73		0.18		0.13		0.53		221.06		312.54		0.13		0.31
			7.45		6.01		1.13		0.13		0.54		331.61		432.95		0.13		0.50
			6.71		12.63		2.14		0.06		0.45		120.29		229.74		0.14		0.62
			7.66		9.32		0.63		0.10		0.49		210.95		311.34		0.14		0.72
			6.78		8.64		1.51		0.09		0.46		310.88		400.51		0.13		0.55
**06**	Yellow River			8.48		92.84		6.26		4.14		2.52		41.27		38.62		2.03	
				7.87		106.76		7.12		4.76		2.90		47.46		44.41		2.33	
**07**	Yinchuan Paddyfield		7.43		102.89		4.89		0.30		0.75		3.45		2.22		0.36		0.49
			7.54		65.35		2.18		0.46		0.38		5.16		3.22		0.27		0.56
			7.34		77.29		1.98		0.48		0.36		5.29		3.45		0.27		0.35
			7.24		98.35		5.61		0.23		0.51		3.08		6.60		0.24		0.22
			7.21		92.60		4.40		0.27		0.67		3.10		2.00		0.32		0.21
			7.45		52.80		1.76		0.37		0.31		4.17		2.60		0.22		0.13
			7.76		88.52		5.05		0.21		0.46		2.77		5.94		0.21		0.14
			7.8		116.27		5.52		0.33		0.84		3.90		2.51		0.41		0.35
**08**	Wuliangsuhai	8.53	8.35	85.00	146.62	21.14	12.41	0.68	2.20	1.13	1.28	10.73	49.73	21.24	59.30	1.93	11.15	0.12	0.34

	Lake		8.60		58.89		8.81		2.61		1.12		46.23		60.87		11.39		0.43
			8.28		147.81		6.82		3.94		1.26		70.03		82.68		11.26		0.49
**09**	Tieshanping River		4.02		25.51		1.38		0.37		1.55		3.09		36.67		0.39		0.52
			4.23		17.57		3.29		0.39		1.58		4.72		37.86		0.39		0.37
			4.12		36.94		1.26		0.19		1.30		0.86		28.48		0.41		0.53
			3.94		18.97		3.55		0.42		1.70		5.09		40.87		0.42		0.60
			3.88		29.42		5.15		0.31		1.55		3.01		35.81		0.43		0.37
			3.91		27.26		4.77		0.29		1.44		2.79		33.17		0.40		0.23
			4.40		39.87		0.76		0.20		1.41		0.93		30.75		0.44		0.22
**10**	Jiamusi Swamp		6.90		25.51		1.38		0.37		1.55		123.09		236.67		0.39		0.40
			6.86		67.58		4.05		1.16		0.95		197.40		211.58		0.36		0.39
			7.59		23.79		2.26		5.72		2.03		120.64		317.59		3.32		0.62
			7.2		25.62		2.29		0.69		0.06		83.39		363.19		1.11		0.70
			6.72		12.63		1.14		0.06		0.45		80.29		129.74		0.14		0.68
			7.47		36.54		1.19		0.18		1.29		170.85		228.18		0.41		0.62
			7.21		29.63		5.02		0.15		1.05		160.69		162.85		0.33		0.49
			7.27		53.52		5.89		0.93		0.00		141.80		210.83		5.52		0.64
**11**	Songhuajiang River	7.27		38.06		3.19		1.09		1.34		11.87		18.58		0.30		0.27	
		7.78		29.83		2.75		0.91		1.31		10.80		27.77		0.24		0.27	
		7.89		32.44		3.13		0.89		1.10		9.63		27.55		0.26		0.19	
**12**	Yanbian Paddyfield		7.28		155.37		7.38		0.45		1.13		125.21		233.36		0.54		0.58
			7.20		98.68		3.29		0.70		0.58		217.80		324.86		0.41		0.75
			6.84		116.71		2.99		0.72		0.54		317.98		425.21		0.41		0.58
			7.18		148.51		8.47		0.35		0.78		234.65		459.96		0.36		0.83
			6.90		139.83		6.64		0.40		1.01		224.69		453.02		0.49		0.63
			6.84		79.73		2.66		0.56		0.47		126.30		393.92		0.33		0.32
			6.84		133.66		7.63		0.32		0.70		214.19		338.96		0.32		0.41
			7.18		175.57		8.34		0.51		1.27		275.88		353.79		0.61		0.46
**13**	Shimen Swamp		8.15		25.24		2.31		0.37		1.53		43.05		186.28		0.39		0.63
			8.22		17.38		3.26		0.38		1.56		44.67		167.45		0.39		0.56
			8.47		36.54		2.19		0.18		1.29		40.85		268.18		0.41		0.73
			8.15		27.24		4.81		0.40		1.65		43.30		139.16		0.42		0.56
			8.22		18.76		3.51		0.42		1.68		55.04		140.43		0.42		0.81
			7.42		39.44		2.68		0.20		1.39		40.92		130.41		0.44		0.61
			8.47		29.10		5.10		0.31		1.54		42.98		235.42		0.43		0.31
			7.49		26.96		1.72		0.28		1.42		42.76		232.81		0.40		0.40
**14**	Antu Reservior		7.76		22.94		3.74		0.34		1.39		2.78		32.98		0.35		0.53
			7.83		15.80		2.96		0.35		1.42		4.24		34.05		0.35		0.38

			8.06		33.22		3.63		0.17		1.17		10.77		25.62		0.37		0.54
			7.76		24.76		2.20		0.36		1.50		3.00		35.60		0.38		0.62
			7.83		17.06		1.19		0.38		1.53		4.58		36.75		0.38		0.39
			7.06		35.85		2.07		0.18		1.26		8.83		27.65		0.40		0.24
			8.06		26.46		0.63		0.28		1.40		2.71		32.20		0.39		0.23
			7.13		24.51		1.29		0.26		1.29		2.51		29.83		0.36		0.14
**15**	Meihekou Paddyfield		7.20		120.17		5.71		0.35		0.87		134.22		222.60		0.42		0.34
			7.40		101.65		5.80		0.24		0.53		174.48		216.82		0.25		0.54
			6.43		95.71		4.55		0.28		0.69		154.41		212.07		0.33		0.62
			7.10		54.57		1.82		0.39		0.32		114.69		232.69		0.23		0.60
			7.01		91.49		5.22		0.22		0.48		149.10		156.14		0.22		0.54
			6.87		67.54		2.25		0.48		0.40		105.75		133.32		0.28		0.43
			7.31		79.89		2.04		0.49		0.37		93.58		153.57		0.28		0.57
			7.23		106.35		5.05		0.31		0.77		131.94		202.30		0.37		0.51
**16**	Changbaishan Peatland		7.50		26.53		0.37		0.71		0.07		389.92		565.45		1.15		0.39
			7.25		29.98		0.68		0.80		0.08		314.61		673.95		1.30		0.56
			8.06		23.06		2.06		0.62		0.06		365.09		556.89		1.00		0.63
			7.23		22.67		2.03		0.61		0.06		462.29		655.92		0.98		0.40
			7.20		25.62		0.29		0.69		0.06		383.39		763.19		1.11		0.25
			7.50		19.71		1.76		0.53		0.05		441.07		748.61		0.85		0.24
			7.68		16.08		1.44		0.43		0.04		315.10		639.66		0.70		0.14
			7.48		18.17		0.63		0.49		0.05		330.07		544.82		0.79		0.16
**17**	Duolun Peatland		7.40		66.01		4.39		1.31		0.12		351.36		521.08		2.12		0.54
			7.76		74.60		4.96		1.48		0.14		397.03		536.82		2.40		0.67
			8.10		57.38		3.82		1.14		0.11		305.41		405.24		1.84		0.78
**18**	Panjin Swamp		8.36		122.60		10.10		1.90		0.12		16.65		68.55		0.26		0.42
			7.86		147.12		12.12		2.28		0.14		17.97		70.26		0.31		0.41
			8.12		98.08		8.08		1.52		0.10		15.31		56.84		0.21		0.65
**19**	Bench-scale wetland																		
**20**	Guilin Paddyfield																		
**21**	Chongqing Paddyfield																		
**22**	Xiamen Paddyfield																		
**23**	Jiaxing																		

	Paddyfield																			
**24**	Yuanmingyuan Lake		8.13	8.02	117.15	86.08	28.00	9.40	0.86	0.99	1.08	1.22	17.40	16.51	19.08	17.66	1.62	1.14	0.23	0.34
**25**	Yongding River			7.59		23.80		2.30		2.00		120.6		120.64		127.59		3.32		0.74
				7.80		20.30		1.30		1.00		75.40		75.44		79.52		5.31		0.72
				7.84		15.60		1.90		1.20		84.1		84.11		111.02		4.32		0.65
**26**	North Canal	Summer	7.89		297.53		7.48		5.66		1.72		90.36		166.88		6.62		0.13	
			7.48		297.53		9.28		3.20		1.72		90.36		176.14		12.03		0.22	
			7.68		213.70		12.12		4.19		2.22		54.04		77.96		2.03		0.28	
			8.08		456.82		10.32		4.31		2.71		49.86		51.24		2.41		0.45	
			7.44		195.36		9.21		5.05		1.48		91.83		117.68		0.90		0.56	
		Winter	7.63		247.94		9.31		3.28		2.19		48.90		118.86		1.07		0.22	
			8.00		326.68		13.57		2.97		1.88		41.56		60.34		3.16		0.21	
			7.56		200.89		11.70		6.09		3.44		66.55		161.40		17.42		0.13	
			8.08		197.01		8.40		4.53		2.50		76.24		124.24		17.79		0.14	
			7.71		225.41		13.18		6.56		3.13		90.46		164.09		17.59		0.35	
**27**	Baiyangdian Lake		7.63	7.56	158.70	241.70	7.90	21.00	2.10	4.60	1.40	1.40	31.30	73.40	12.07	54.33	0.69	5.38	0.19	0.52
			7.60	8.26	209.10	241.70	14.80	21.00	1.90	2.60	1.20	1.40	26.60	73.40	38.62	61.85	2.03	9.78	0.24	0.68
			7.56	7.68	256.60	173.60	18.80	18.10	3.90	3.40	2.20	1.80	42.60	43.90	39.30	63.33	11.15	1.65	0.18	0.61
			8.51	7.65	126.10	371.10	10.90	13.00	2.90	3.50	1.60	2.20	48.80	40.50	79.52	41.63	11.39	1.96	0.29	0.79
			7.71	7.68	336.30	158.70	14.60	20.40	4.20	4.10	2.00	1.20	57.90	74.60	41.02	14.36	11.26	0.73	0.14	0.61
**28**	Shangqiu Reservior			7.63		121.87		12.58		1.39		1.22		17.54		27.09		2.01		0.16
				7.66		108.52		11.20		1.24		1.08		15.62		24.12		1.79		0.26
**29**	Changshu Paddyfield			6.90		260.14		9.61		0.75		1.88		38.72		75.62		0.91		0.66
				6.84		148.33		3.85		1.05		0.87		41.72		77.30		0.62		0.51
				7.18		276.28		12.26		0.66		1.45		48.66		58.53		0.67		0.73
				7.00		183.58		4.76		1.30		1.08		44.50		59.03		0.77		0.55
				7.20		217.13		4.32		1.34		1.01		34.85		69.70		0.75		0.28
				7.10		248.66		11.04		0.59		1.30		47.79		76.68		0.60		0.36
				7.40		326.62		12.06		0.94		2.37		30.95		67.06		1.14		0.41
**30**	Jiaxing constructed wetland	Winter	8.48		67.80		16.70												0.29	
			8.35		50.00		14.10												0.24	
			8.60		90.90		7.40												0.28	
			8.28		94.30		24.70												0.31	
			8.53		85.80		34.10												0.34	
			7.63		72.70		9.90												0.37	

		Summer	8.02		83.20		21.70												0.21	
			8.13		75.20		16.80												0.24	
			7.40		65.40		12.60												0.23	
			7.50		62.10		23.30												0.21	
		Waterward		8.10		61.00		8.70												
				8.20		69.80		18.50												
				7.60		63.10		14.40												
				8.10		55.00		10.90												
				8.10		52.30		19.80												
**31**	Chaohu Lake		6.63	6.93	328.40	184.60	27.30	10.80	0.94	1.30	2.40	1.10	11.01	14.58	17.09	19.08	1.15	0.77	0.27	0.88
			7.54	6.59	313.90	218.30	31.30	9.80	0.75	1.35	1.60	1.00	9.84	14.93	21.05	19.75	0.76	0.76	0.20	0.66
			8.00	7.45	260.72	410.49	11.22	34.11	1.84	1.18	1.53	2.97	20.60	13.76	22.83	18.87	1.09	1.43		
			7.70	8.40	308.35	392.36	13.80	39.18	1.90	0.93	1.44	2.05	21.08	12.30	23.77	26.32	1.07	0.95		
			7.70	7.30	210.65	369.44	12.29	30.70	1.49	1.06	1.24	2.68	16.64	12.38	20.36	17.98	0.88	1.29		
			7.60	7.30	249.14	353.13	7.15	35.26	1.54	0.84	1.16	1.85	17.04	11.07	31.13	33.68	0.86	0.85		
			8.00	7.90	395.23	463.85	23.07	38.55	2.79	1.33	2.33	3.36	31.23	15.55	39.45	20.02	1.65	1.62		
**32**	Poyang Lake			6.86		67.60		7.10		1.16		1.00		17.40		41.58		0.36		0.58
				7.38		84.90		9.70		1.33		0.80		9.42		46.96		0.34		0.41
				6.88		99.40		8.40		1.33		1.10		13.76		48.87		0.32		0.59
**33**	Dongting Lake		8.13	7.77	142.70	53.50	27.00	5.90	1.67	0.93	1.00	0.00	7.57	41.80	9.08	70.83	0.44	5.52	0.10	0.67
				7.92		83.40		5.00		0.75		0.00		50.10		89.40		1.61		0.42
**34**	Hunan Paddyfield			6.80		305.88		14.53		0.88		2.22		70.25		96.61		1.07		0.15
				7.63		292.37		16.68		0.69		1.53		39.16		79.61		0.71		0.25
				6.90		275.29		13.07		0.79		1.99		39.23		75.95		0.96		0.32
				6.84		263.14		15.01		0.62		1.38		28.25		87.65		0.64		0.51
				7.18		345.64		16.41		0.99		2.50		51.58		77.47		1.21		0.63
				7.28		194.27		6.48		1.37		1.14		55.35		79.56		0.81		0.74
				7.20		229.77		5.88		1.42		1.07		45.71		90.26		0.80		0.48
				7.40		156.97		5.23		1.11		0.92		32.40		117.72		0.66		0.34
**35**	Pearl River	Summer	7.63		158.70		15.80		3.51		1.40		31.30		65.11		5.01		0.11	
			7.77		252.10		9.90		4.13		2.00		41.80		70.83		5.52		0.15	
			7.92		214.20		11.30		2.15		1.60		30.10		40.40		1.61		0.23	
		Winter	7.56		161.10		6.50		1.96		1.40		73.40		82.98		3.71		0.22	
			7.72		139.40		13.10		3.75		1.20		26.60		35.08		4.55		0.17	
			7.78		229.50		14.70		2.99		1.60		48.80		63.64		5.68		0.19	

Table 3 Spearman correlation matrix between anammox rates and the physicochemical parameters

			Moisture	Total organic matter	pH	NH_4^+	NO_X^-	Total nitrogen	Total phosphorus	Total carbon	Total sulfur	DO
Anammox rates	Anammox rates	r	0.284**	-0.019	0.369**	0.243**	0.583**	0.280**	0.130	0.139	0.217**	-0.238**
		p	0.000	0.798	0.000	0.001	0.000	0.000	0.086	0.066	0.004	0.002
		n	192	192	182	182	182	177	177	177	177	167
	River	r	0.222	0.357*	-0.012	0.593**	0.695**	0.384*	0.379*	0.077	0.343*	-0.513**
		p	0.180	0.028	0.942	0.000	0.000	0.017	0.019	0.647	0.035	0.001
		n	38	38	38	38	38	38	38	38	38	38
	Lake	r	0.090	-0.077	0.045	0.266	0.626**	0.082	0.351*	0.119	0.040	-0.684**
		p	0.572	0.629	0.776	0.089	0.000	0.605	0.023	0.451	0.803	0.000
		n	42	42	42	42	42	42	42	42	42	42
	Paddyfield	r	-0.036	0.360*	0.031	0.346*	0.226	0.314	0.241	0.633**	0.238	-0.065
		p	0.828	0.024	0.851	0.031	0.167	0.052	0.139	0.000	0.145	0.694
		n	39	39	39	39	39	39	39	39	39	39
	Constructed wetland	r	-0.227		-0.118	0.307	0.621*					-0.360
		p	0.275		0.675	0.265	0.013					0.307
		n	25		15	15	15					10
	Reservoir	r	0.248	0.697*	-0.318	0.333	0.261	0.709*	-0.164	-0.152	0.555	-0.430
		p	0.489	0.025	0.370	0.347	0.467	0.022	0.651	0.676	0.096	0.214
		n	10	10	10	10	10	10	10	10	10	10
	Swamp	r	0.315	-0.171	0.129	0.284	0.438**	0.198	0.232	-0.372*	-0.121	0.230
		p	0.054	0.304	0.439	0.084	0.006	0.235	0.161	0.021	0.470	0.165
		n	38	38	38	38	38	38	38	38	38	38

** Correlation is significant at the 0.01 level (2-tailed); * Correlation is significant at the 0.05 level (2-tailed).

Table 4 Spearman correlation matrix between anammox rates and some biogeographic parameters

			Altitude	Precipitation	Average temperature	Diurnal temperature range	Sunshine time
Anammox rates	Anammox rates	r	-0.446**	0.195**	0.332**	-0.304**	-0.018
		p	0.000	0.007	0.000	0.000	0.804
		n	192	192	192	192	192
	River	r	-0.555**	0.400*	0.199	0.008	0.024
		p	0.000	0.013	0.231	0.961	0.885
		n	38	38	38	38	38
	Lake	r	-0.189	-0.176	-0.090	0.053	0.159
		p	0.232	0.264	0.570	0.740	0.313
		n	42	42	42	42	42
	Paddyfield	r	-0.416**	0.364*	0.508**	-0.364*	-0.359*
		p	0.008	0.023	0.001	0.023	0.025
		n	39	39	39	39	39
	Constructed wetland	r	0.204	-0.204	-0.204	0.204	0.204
		p	0.328	0.328	0.328	0.328	0.328
		n	25	25	25	25	25
	Reservoir	r	-0.696*	0.696*	0.696*	-0.696*	-0.696*
		p	0.025	0.025	0.025	0.025	0.025
		n	10	10	10	10	10
	Swamp	r	-0.639**	0.463**	-0.132	-0.590**	0.118
		p	0.000	0.003	0.431	0.000	0.481
		n	38	38	38	38	38

** Correlation is significant at the 0.01 level (2-tailed); * Correlation is significant at the 0.05 level (2-tailed).

Table 5 Equations used for the estimated budget of N loss by anammox in China inland waters and wetland ecosystem[a]

#	Item	Equation	Explanation
		**Total N loss in various types of inland waters and wetland ecosystem**	
i	The area of various types of wetlands in China (A)[b]	A = sum of the area of various types of wetlands in China	Total area of Paddy field was according to the data

			from IRRI (IRRI, 2009);<br>The total area of River, Swamp, Lake, Constructed wetland, and Artificial pond were based on Landsat and CBERS-02B remote sensing data (Niu 2012).
	The area of interface ($A_{Interface}$)[c]<br>of river<br><br>of lake and artificial pond	$A_{Interface}$ = the total length of river in China (~430,000 km) × the average width of interface (~1 m) × sides (2)<br>$A_{Interface}$ = total perimeter of lake and artificial pond in China (~56,000 km and ~15,000 km) × the average width of interface (~1 m)	For River, Lake and Artificial pond, these data were estimated based on the interface research over the past 20 years by Research Center for Eco-Environmental Sciences, Chinese Academy of Sciences (Yin 1995, 1995, 2006; Wang 2006; Wang 2010; Wang 2012; Zhu 2013)
	The area of open water ($A_{Open\ water}$)	$A_{Open\ water} = A - A_{Interface}$	
ii	Average activity [d, e]<br>of anammox (C)<br><br>of denitrification (E)	C = the average of anammox rates in corresponding type or zone of sampling wetlands<br>E = the average of denitrification rates in corresponding type or zone of sampling wetlands	The rates of anammox and denitrification were measured using ^{15}N isotopic tracing method, shown in Fig 1c, Fig 2b, Fig 3.
iii	Total N loss in China [d, e]<br>by anammox (D)<br><br>by denitrification (F)	D = C × A; In particularly,<br>$D_{River,\ Lake,\ and\ Artificial\ pond} = D_{Interface} + D_{Open\ water}$<br>F = E × A; In particularly,<br>$F_{River,\ Lake,\ and\ Artificial\ pond} = F_{Interface} + F_{Open\ water}$	Estimated N budget by anammox<br><br>Estimated N budget by denitrificaiton
iv	Contribution of anammox to Total N loss (G)	G = (D/(D+F)) × 100%	The percent of N loss by anammox to anammox plus denitrification

Total N loss in China inland waters and wetland ecosystem

v	Total N loss[4, 5]		
	by anammox (D_{total})	D_{total} = sum (D)	Reach to 2.0 Tg yr^{-1}
	by denitrification (F_{total})	F_{total} = sum (F)	~ 15.0 Tg yr^{-1}
vi	Contribution of anammox to Total N loss (G_{total})	$G_{total} = (D_{total}/(D_{total}+F_{total}))$ × 100%	Up to 11.4 %

[a] The number of samples. We have more than two hundred sampling sites, yet it is still limited to estimate the emission budgets of China, especially of inland waters with high heterogeneity. However, it is a little hard to determine how much samples are enough to estimate the emission budgets covering entire China;
[b] The influence of spatio-temporal change and seasonal drought/flood on the area of inland water are not discussed;
[c] The accurate area of hotspot, the heterogeneous and homogeneous area of different inland water are also not determined;
[d] The affection of seasons and temperature on the anammox rate are not discussed. The samples about the temporal anammox rate are very limited;
[e] The paddyfield is the largest contributor of anammox in different inland waters, but whether the anammox rates in different rice growing season also are constant is still not known.

Table 6 Spearman correlation between anammox abundance and N_2O flux emission

		N_2O flux emission
Anammox abundance	r	-0.877^{**}
	p	0.000
	n	20

** Correlation is significant at the 0.01 level (2-tailed).

Table 7 Equations used for the calculation of anammox and denitrification in intact cores

#	Equation	Explanation
1	$A_{total} = F_N^{-1} \times [P^{29} N_2 + 2 \times (1-F_N^{-1}) \times P^{30} N_2]$	N_2 production by anammox in anoxic slurry assays
2	$D_{total} = P^{30} N_2 \times F_N^{-2}$	N_2 production by denitrification in anoxic slurry assays
3	$r_{14} = [(1-ra) \times (P^{29} N_2 / P^{30} N_2) - ra] / (2-ra)$	Caculation of r_{14} based on ra
4	$TN_2P = 2r_{14} [P^{29} N_2 + P^{30} N_2 (1 - r_{14})]$	Total N_2 production in intact cores
5	$A_{total\text{-}in\ situ} = 2r_{14} \times (P^{29} N_2 - 2r_{14} \times P^{30} N_2)$	N_2 production by anammox in intact cores
6	$D_{total\text{-}in\ situ} = TN_2P - A_{total\text{-}in\ situ}$	N_2 production by denitrification in intact cores

$P^{29}N_2$ and $P^{30}N_2$ are rates of production of $^{29}N_2$ and $^{30}N_2$ in the $^{15}NO_3^-$ treatment; F_N is the fraction of ^{15}N in NO_3^-.

Table 8 Primers used in this study and correspondence thermal profiles

Specificity	Primer	Sequence (5'-3')	Thermal profiles	Reference
Planctomycetales (PCR)	pla46f	GGATTAGGCATGCAAGTC	5 min at 94 ℃, 30 cycles consisting of 1 min at 94 ℃, 1 min at 50 ℃ and 2 min at 72 ℃.	Juretschko et al. 1998; Neef et al., 1998
	630r	CAKAAAGGAGGTGATCC		
Anammox 16S rRNA (PCR)	Amx368f	TTCGCAATGCCCGAAAGG	10 min at 96 ℃, 30 cycles consisting of 1 min at 96 ℃, 1 min at 52 ℃ and 1 min at 72 ℃.	Schmid MC, et al. 2005
	Amx820r	AAAACCCCTCTACTTAGTGCCC		
Anammox *hzsA* (qPCR)	hzsA_1597F	WTYGGKTATCARTATGTAG	3 min at 96 ℃, 40 cycles consisting of 30 s at 96 ℃, 30 s at 55 ℃, and 30 s at 72 ℃	Harhangi et al. 2012
	hzsA_1857R	AAABGGYGAATCATARTGGC		
Anammox *hzsB* (qPCR)	HSBeta396F	ARGGHTGGGGHAGYTGGAAG	3 min at 95 ℃, 40 cycles consisting of 30 s at 95 ℃, 30 s at 59 ℃ and 30 s at 72 ℃.	Wang et al. 2012
	HSBeta742R	GTYCCHACRTCATGVGTCTG		

Supplementary references

Thompson JD, Gibson TJ, Plewniak F, Jeanmougin F, Higgins DG. (1997). The CLUSTALX windows interface: flexible strategies for multiple sequence alignment aided by quality analysis tools. *Nucleic Acids Res* **25:** 4876–4882.

Schloss PD, Handelsman J. (2005). Introducing DOTUR, a computer program for defining operational taxonomic units and estimating species richness. *Appl Environ Microbiol* **71:** 1501–1506.

Tamura K, Dudley J, Nei M, Kumar S. (2007). MEGA4: molecular evolutionary genetics analysis (MEGA) software version 4.0. *Mol Boil Evol* **24:** 1596–1599.

Bao SD. (ed.). (2000). Chemical Analysis for Agricultural Soil. China Agriculture Press: Beijing.

Gundersen JK, Ramsing NB, Glud RN. (1998). Predicting the signal of O_2 microsensors from physical dimensions, temperature, salinity, and O_2 concentration. *Limnol Oceanogr* **43:** 1932–1937.

Liu D, Ge Y, Chang J, Peng C, Gu B, Chan GYS *et al.* (2009). Constructed wetlands in China: recent developments and future challenges. *Front Ecol Environ* **7:** 261–268.

International Riee Research Institute (IRRI). IRRI World Rice Statistics (http://www.irri,org/) (DB/OL).
Niu Z G, Zhang H Y, Wang X W, et al. Mapping wetland changes in China between 1978 and 2008. Chin Sci Bull, 2012, 57(22), 2813–2823.
Yin C. The ecological function, protection and utilization of land/inland water ecotones. Acta Ecologica Sinica 1995, 15(3):331-335.
Yin C, Lan Z. The nutrient retention by ecotone wetlands and their modification for Baiyangdian lake restoration[J]. Water Science & Technology, 1995, 32(3):159–167.
Yin C, Arheimer B, Verhoeven J T A, et al. Regional and global concerns over wetlands and water quality[J]. Trends in Ecology & Evolution, 2006, 21(2): 96-103.
Wang H, Wang W, Yin C, et al. Littoral zones as the “hotspots” of nitrous oxide (N2O) emission in a hyper-eutrophic lake in China[J]. Atmospheric Environment, 2006, 40(28):5522–5527.
Wang L, Yin C, Wang W. Sedimentary Enzyme Kinetics of Land/Water Ecotones with Reed Domination[J]. CLEAN-Soil, Air, Water, 2010, 38(2):194-201.
Wang S, Zhu G, Peng Y, et al. Anammox bacterial abundance, activity, and contribution in riparian sediments of the pearl river estuary[J]. Environmental Science Technology, 2012, 46(16): 8834-8842.
Zhu, G. B., Wang, S. Y., Wang, Y., Wang, C. X., Risgaard–Petersen, N., Jetten, M. S. M., Yin, C. Q., 2011. Anaerobic ammonia oxidation in a fertilized paddy soil. ISME J. 5 (12), 1905–1912.
Zhu, G., Wang, S., Wang, W., Wang, Y., Zhou, L., Jiang, B., Op den Camp, H. J. M., Risgaard–Petersen, N., Schwark, L., Peng, Y., Hefting, M. M., Jetten, M. S. M., Yin, C., 2013. Hotspots of anaerobic ammonium oxidation at land–freshwater interfaces. Nature Geosci. 6 (2), 103–107.

The detailed information of sampling sites

- **Bosten Lake**

Bosten Lake (41°49'-41°54' N; 86°43'-86°57' E) is located in the Xinjiang Uygur Autonomous Region, which is the largest inland freshwater lake in China (supplementary Fig. S7). The lake, which is about 55 km long from east to west and 25 km wide from north to south, covers an area of 1,100 km^2. Bosten Lake receives inflow water from a catchment area of 56,000 km^2. The elevation is 1,048 m above sea level and the water depth ranges from 0.8 to 17 m with an average depth of 9 m.

Bosten Lake is a tectonic lake formed by tectonic subsurface interactions. The average influent and effluent is 2.68 and 1.25 billion m^3, respectively. The lake water discharges through the Kongque River in the southwest corner. The mineralization degree of Bosten Lake is maintained at about 1.3 g / L.

The annual average atmospheric temperature is 7.9 ℃. In January, the average atmospheric temperature is -12.7 ℃ with the extreme lowest temperature of -30.2 ℃ and the average water temperature is 0.6 ℃. In July, the average atmospheric temperature is 22.8 ℃ with the extreme highest temperature of 38 ℃ and the average water temperature is 23 ℃.

Sediment samples were collected in the littoral zone (about 2 m from the water / land interface) of Bosten Lake.

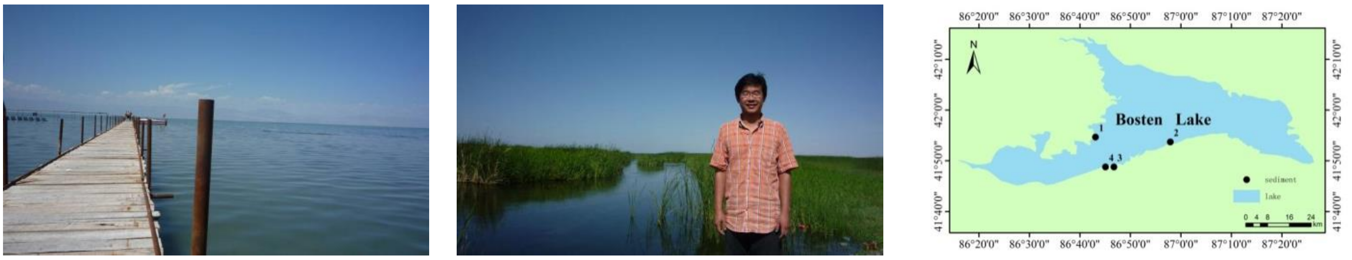

Figure S7. Landscape and geographical distribution of the sampling site and the author Dr. Guibing Zhu in Bosten Lake. The photograph was taken by author Shanyun Wang with the permission of Dr. Zhu. The map were come from web of "Data Sharing Infrastructure of Earth System Science" http://www.geodata.cn. All of the maps used in the manuscript are free. The geographical distribution of sampling sites was drawn with software ArcGIS.

- **Tarim River**

Tarim River (41°03'-41°04' N; 86°06'-86°07' E) with a length of 2,421 km, which is located in the northern Tarim Basin of Xinjiang Uygur Autonomous Region, is the longest inland river in China and the fifth largest inland river in the world (supplementary Fig. S8).

The area of the Tarim River Basin is about 435,500 km^2. The annual runoff is 39.83 billion m^3 and the water supply is mainly from snowmelt of glacier. The non-repetitive groundwater resources are 3.07 billion m^3 and the total water resources are 42.9 billion m^3. It is a continental warm temperate

zone with extremely arid desert climate, with characteristic of extremely scanty precipitation, intensive evaporation, great daily temperature difference, heavy silt load, plentiful sunshine and heat resources in Tarim Basin. The annual average temperature is around 10.6 to 11.5 ℃. In July, the average temperature is 20 to 30 ℃ and the extreme highest temperature is 43.6 ℃. In January, the average temperature is -10 to -20 ℃ and the extreme lowest temperature is -30.9 ℃. The annual average precipitation is 17.4 to 42.8 mm and annual average evaporation is 1,125 to 1,600 mm. The samples were collected in the riparian zone (about 2 m from the water / land interface) of Tarim River close to Koria city.

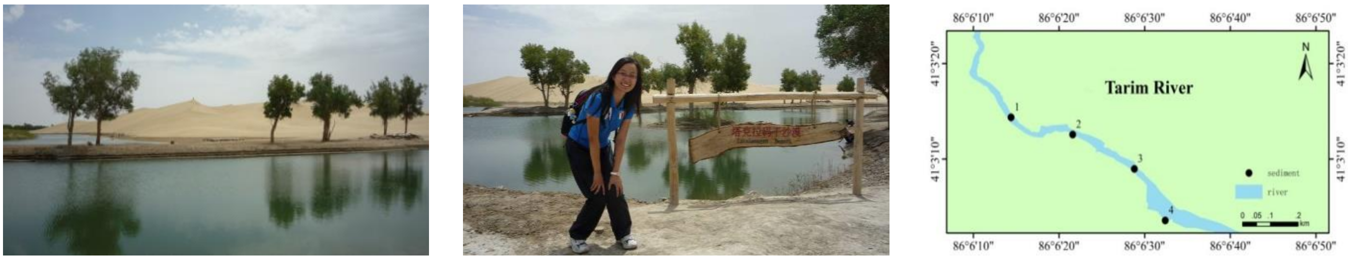

Figure S8. Landscape and geographical distribution of the sampling site and the author Dr. Shanyun Wang in Tarim River. The photograph was taken by author Guibing Zhu with the permission of Dr. Wang. The map were come from web of "Data Sharing Infrastructure of Earth System Science" http://www.geodata.cn. All of the maps used in the manuscript are free. The geographical distribution of sampling sites was drawn with software ArcGIS.

- **Tianchi Lake**

Tianchi Lakes (43°54' N; 88°08' E) in Tianshan Mountains lies on the north hillside of Mount Bogda (5,445 m) which is the peak in the eastern part of Tianshan Mountains in Xinjiang Uygur Autonomous Region of China (supplementary Fig. S9).

Tianchi Lake is typical natural high altitude (1,928 m) freshwater lakes. The lakes are 3,500 m long from north to south, 800 to 1,500 m wide from east to west. The lakes area is 4.9 km^2 with the maximum lake depth of about 103 m. The main water source of Tianchi Lakes is glacier and snow melted water. The annual average air temperature is 3 to 4 ℃, and the total water storage capacity is 200 million m^3.

The sample was taken in the littoral zone (about 5 m from the water / land interface) of the main lake of Tianshan Lakes.

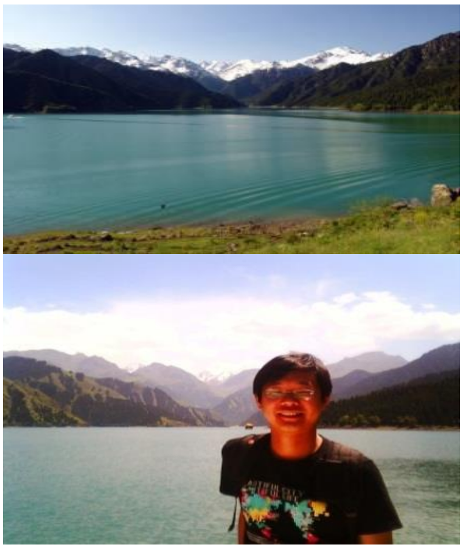

Figure S9. Landscape and Dr. Guibing Zhu in Tianchi Lakes. The photograph was taken by author Shanyun Wang with the permission of Dr. Zhu. The map were come from web of "Data Sharing Infrastructure of Earth System Science" http://www.geodata.cn. All of the maps used in the manuscript are free. The geographical distribution of sampling sites was drawn with software ArcGIS.

- **Poyang Lake**

Poyang Lake (28°22'-29°45' N; 115°47′-116°45′ E) is located in northern Jiangxi Province. As a tectonic lake, Poyang Lake is the largest freshwater lake and the second largest lake in China (supplementary Fig. S10). It is 173 km long from north to south and 50-70 km wide from east to west. The narrow part in the north of the lake is only 5 to 15 km long and the average width of the lake is 16.9 km. The length of the lake shoreline is 1,200 km.

The annual average water level is 12.86 m. The area of the lake surface is 4,070 km^2 and the water storage capacity is 30 billion m^3 maximumly. When the lake is at its lowest water level of 5.9 m, the area of the lake surface is only 146 km^2 and the water storage capacity is 450 million m^3. The area of Poyang Lake basin is 162,200 km^2. Poyang Lake is the largest wintering area for swans and white cranes (accounting for more than 98% of the wintering white cranes in the world). Hundreds of thousands of swans return here every year. The sediment samples were collected in the Jiujiang section of Poyang Lake.

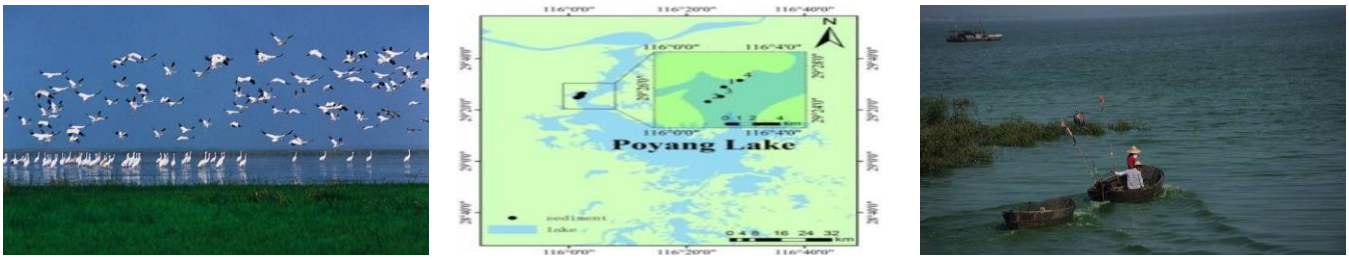

Figure S10 Landscape and geographical distribution of the sampling site in Poyang Lake. The photograph was taken by author Guibing Zhu. The map were come from web of "Data Sharing Infrastructure of Earth System Science" http://www.geodata.cn. All of the maps used in the manuscript are free. The geographical distribution of sampling sites was drawn with software ArcGIS.

- **Dongting Lake**

Dongting Lake (28°30'-30°20' N; 110°40′-113°10′ E), as the second largest freshwater lake in China, is mainly located in northern Hunan Province (supplementary Fig. S11). The average altitude of the lake is 33.5 m and the maximum depth is 30.8 m with an average depth of 6-7 m. The area of the lake region and the lake surface is 2,820 and 4,040 km^2, respectively. The water storage of the lake is 17.8 billion km^3.

The annual average temperature of Dongting Lake is 16.4 to 17 ℃. In January, the average temperature is 3.8 to 4.5 ℃ with the lowest temperature of -18.1 ℃. The frost-free period in lake region is 258 to 275 d and the annual precipitation is 1000 to 1400 mm. As a major lake of Yangtze River Basin, the annual average runoff of Dongting Lake is 201.6 billion km^3, which approximately accounts for 21% of the surface water in Yangtze River.

The sampling site was located at the Yueyang section in Hunan Province. Soil and sediment samples were collected in the littoral zone (1 m and 3 m from the water / land interface).

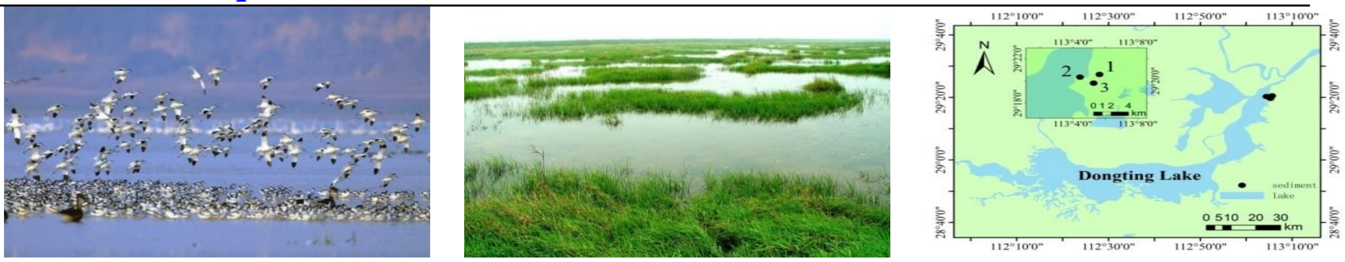

Figure S11. Landscape and geographical distribution of the sampling site in Dongting Lake. The photograph was taken by author Guibing Zhu. The map were come from web of "Data Sharing Infrastructure of Earth System Science" http://www.geodata.cn. All of the maps used in the manuscript are free. The geographical distribution of sampling sites was drawn with software ArcGIS.

- **Turpan river**

Turpan is located in the middle east part of Xinjiang Uygur Autonomous Region (41°12'-43°40' N; 87°16'-91°55' E). It is 240 km long from north to south and 300 km wide from east to west of Turpan area (supplementary Fig. S12).

It is a typical continental arid desert climate in Turpan. Although the annual average temperature is 14.5 ℃, there are more than 100 days per year with a temperature higher than 35 ℃ and 38 days per year with a temperature higher than 38 ℃.The annual average precipitation is only 16 mm.

Sampling site was located at the Toyukmazar Grand Canyon. The mountains of the canyon are more than 100 km long and about 500 m high on average with the peak height of 831.7 m. The highest temperature is 47.8 ℃ in summer and the highest surface temperature is above 70 ℃ at the sampling site, which is the hottest temperature in China.

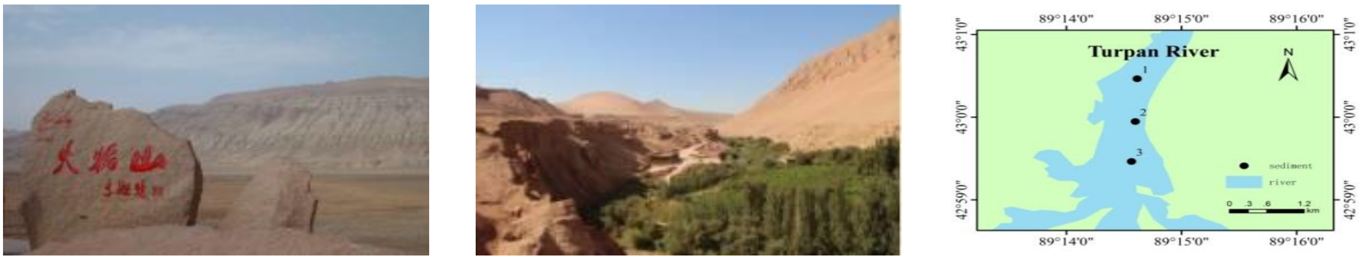

Figure S12. Landscape and geographical distribution of the sampling site in Turpan River. The photograph was taken by author Guibing Zhu. The map were come from web of "Data Sharing Infrastructure of Earth System Science" http://www.geodata.cn. All of the maps used in the manuscript are free. The geographical distribution of sampling sites was drawn with software ArcGIS.

- **The Yellow River**

The Yellow River is the second longest river in China and the fifth longest river in the world (supplementary Fig. S13). It originates from Qinghai Province and flows through nine provinces before it empties into the Bohai Sea. It has an east-west extent of 1,900 km and a north-south extent of 1,100 km of Yellow River. The overall length is about 5,464 km with the basin area of 795,000 km^2. The altitude of the estuary is 4,830 m lower than that of the river source.The annual average discharge of the Yellow River Basin is about 1,775 m^3 / s and the annual average runoff is 58 billion m^3 with the

annual average runoff depth of 77 mm. The water resource of the basin is 593 m^3 / capita and the unit-area average irrigation water consumption is 5410 m^3 / ha. The maximum width of river mouth area is 1,500 m, most of which are around 500 m and the narrowest section is only 50 m. The water depth in the river mouth is around 2.6 m and only 1.2 to 1.6 m for minimum. In the basin area, the percent of rocky mountainous area, hilly and loess area, windy desert area and plain area are 29 %, 46 % , 11 % and 14 %, respectively. The sediment sample was collected under the Yellow River Bridge of Lanzhou section.

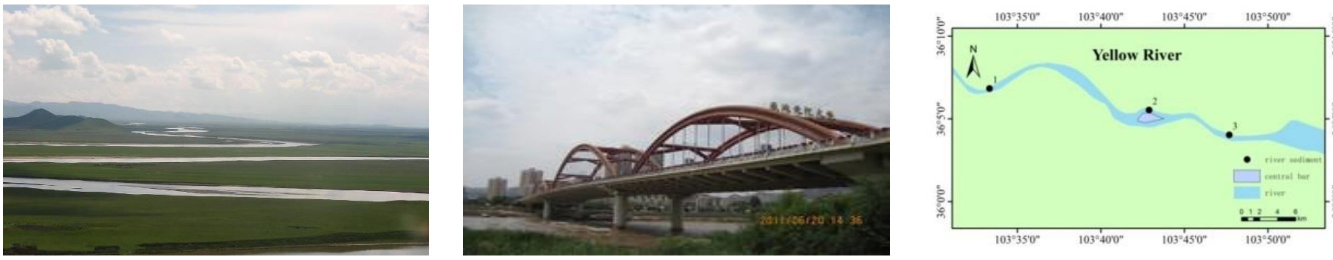

Figure S13. Landscape and geographical distribution of the sampling site in Yellow River. The photograph was taken by author Guibing Zhu. The map were come from web of "Data Sharing Infrastructure of Earth System Science" http://www.geodata.cn. All of the maps used in the manuscript are free. The geographical distribution of sampling sites was drawn with software ArcGIS.

- **Ulansuhai Nur**

Ulansuhai Nur, which located in the Bayannur city of Inner Mongolia, is one of the eight largest freshwater lakes in China (supplementary Fig. S14). Ulansuhai Nur is a large scale grassland lake which is scarcely located in a desert and semidesert region. The lake is the largest wetland in the same latitude on earth. The area of the lake is about 293 km^2, with an altitude of 1,018.5 m. The lake has a north-south extent of 35 to 40 km and an east-west extent of 5 to 10 km. The depth of the lake is 0.5 to 1.5 m with the maximum depth of 4 m. The water retention capacity is 2,500 to 3,000 million m^3.

The formation of Ulansuhai Nur is caused by the diversion of Yellow River, which is so called furiotile-lake. Recent years, large amount of nutrient was poured into the lake. The scale of macrophytic enlarged year by year and Ulansuhai Nur was turned into a grassland lake. Therefore, this young lake has showed an aging trend. The thickness of upper layer sediment which is constitute of lithologic silt sandy loam is 0.2 to 0.5 m. It appears to be black-gray colour with offensive odor. The particle composition is mainly fine-sand. The deeper part is light yellow original soil. Both the riparian sediments and waterward sediments were sampled in middle area of Ulansuhai Nur.

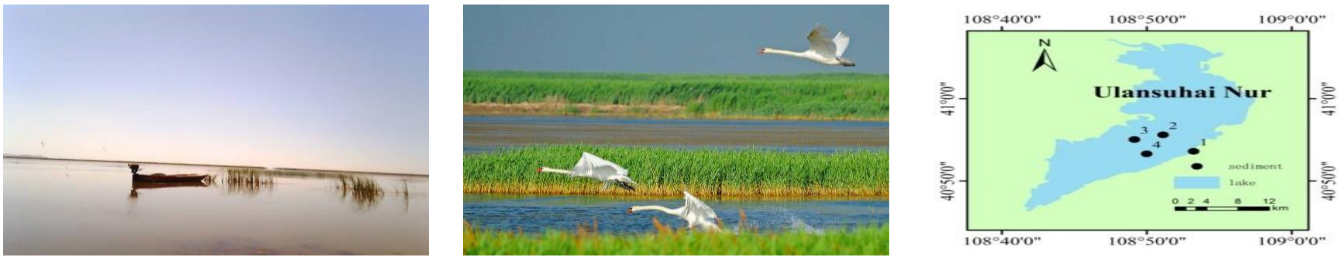

Figure S14. Landscape and geographical distribution of the sampling site in Ulansuhai Nur. The photograph was taken by author Guibing Zhu. The map were come from web of “Data Sharing Infrastructure of Earth System Science” http://www.geodata.cn. All of the maps used in the manuscript are free. The geographical distribution of sampling sites was drawn with software ArcGIS.

- **The Old Summer Palace**

The Old Summer Palace is located in Beijing city, the capital of China. It was built in 1707 in Qing Dynasty and the total area is 350 ha with the lake area of 140 ha (supplementary Fig. S15). It is a royal garden built by the emperors of Qing Dynasty and is a famous attraction in Beijing. The Old Summer Palace Lake is a typical city lake, the water supply is mainly from city rivers. The sediment samples were collected from the riparian zone of the lakes in Old Summer Palace.

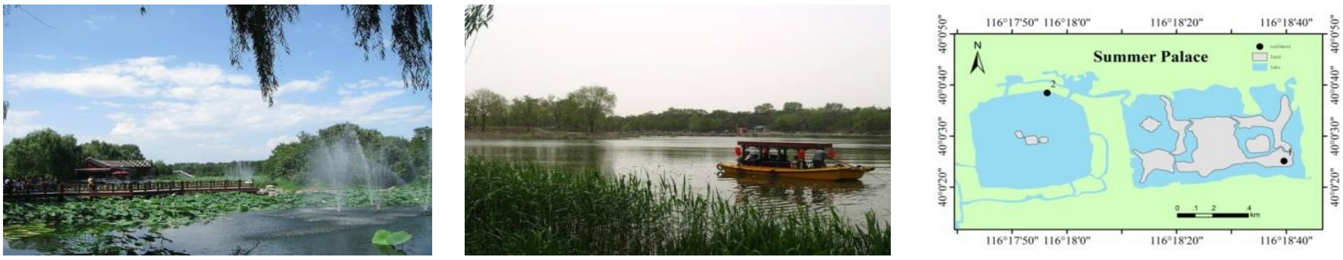

Figure S15. Landscape and geographical distribution of the sampling site in Summer Palace Lake. The photograph was taken by author Guibing Zhu. The map were come from web of “Data Sharing Infrastructure of Earth System Science” http://www.geodata.cn. All of the maps used in the manuscript are free. The geographical distribution of sampling sites was drawn with software ArcGIS.

- **Songhuajiang River**

The Songhuajiang River Basin is located in the north part of northeastern China (supplementary Fig. S16). The river originated from the Tianchi Lake in Changbai Mountain at the border of China and North Korea. It has an east-west extent of 920 km and a north-south extent of 1,070 km and the total length of the river is 1,927 km, which is the largest tributary in the right bank of Heilong River. The basin area is 556,800 km^2 accounting for 30.2% of the Heilongjiang Basin. The runoff is 75.9 billion m^3 which is more than that of the Yellow River.

The Songhuajiang River Basin locates in the north temperate monsoon climate zone with large temperature difference within one year. The annual average temperature is 3 to 5 ℃. The highest temperature occurs in July at 20 to 25 ℃ on average and the highest temperature in history could reach more than 40 ℃. The lowest temperature was lower than -20 ℃ in January. The sediment sample was taken from the riparian zone of both sides of Songhuajiang River in Harbin section.

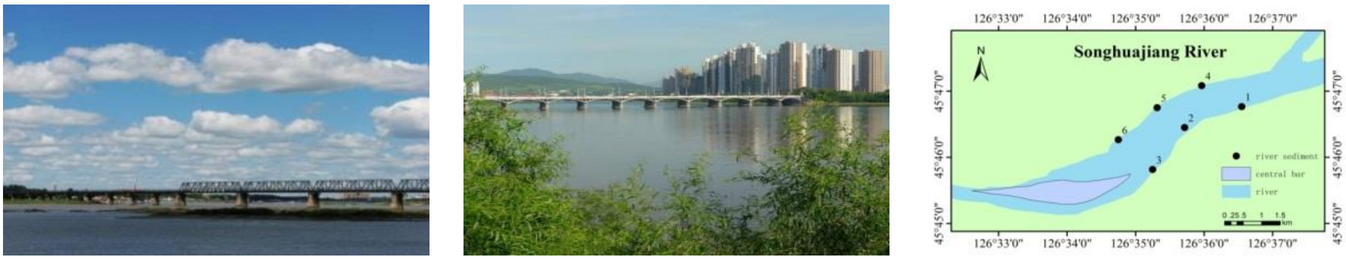

Figure S16. Landscape and geographical distribution of the sampling site in Songhuajiang River. The photograph was taken by author Guibing Zhu. The map were come from web of “Data Sharing Infrastructure of Earth System Science” http://www.geodata.cn. All of the maps used in the manuscript are free. The geographical distribution of sampling sites was drawn with software ArcGIS.

- **Chaohu Lake**

Chaohu Lake is located in the middle of Anhui province, which is one of the five largest freshwater lakes in China (supplementary Fig. S17). The Lake has an east-west extent of 21 km and a north-south extent of 54.5 km. The total lake shoreline length is 184.66 km and the insulosity of the Lake is 0.13 %. The lake area is 753 to 774 km^2 with the water resources of 1.72 to 3.23 billion m^3 when the water depth was 8 to 10 m. Chaohu Lake is a tectonic lake and has a north subtropical monsoon climate. The annual average temperature of the lake basin is 16.1 ℃. The average temperature in January is 2.7 ℃ while 28.7 ℃ in July. The annual average sunshine hours are 2170.1 h and the percentage of sunshine is 49.0 %. The frost-free period is 263 d. The number of precipitation days is 120 d and the precipitation is 998.7 mm. Most of the precipitation which is 535.1 mm for average occurs in May to September. The annual average evaporation capacity is 1,124.4 mm. The ice period is about 20 d and most of the ice appears at the bank area. The sediment samples from the Chaohu Lake were collected from both riparian zone and open water area for comparison.

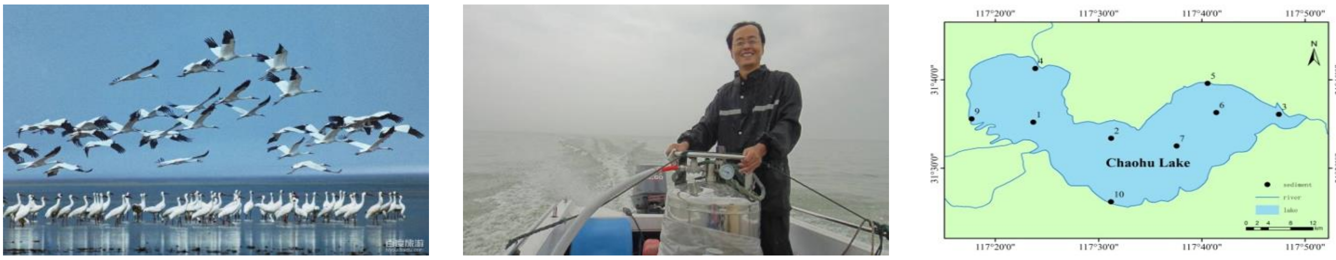

Figure S17. Landscape and geographical distribution of the sampling site and the workmate Dr. Wenqiang Zhang in Chaohu Lake. The photograph was taken by author Guibing Zhu with the permission of Dr. Zhang. The map were come from web of “Data Sharing Infrastructure of Earth System Science” http://www.geodata.cn. All of the maps used in the manuscript are free. The geographical distribution of sampling sites was drawn with software ArcGIS.

- **Yongding River**

The Yongding River located in southwest Beijing is the longest river in Beijing (supplementary Fig. S18). It is 650 km long and the basin area is 50,500 km^2 (45,063 km^2 for mountain area and 1,953 km^2 for plain area). It flows through Shanxi, Hebei province and Beijing city and empties at Tianjin city into Haihe River. The main tributaries of Yongding River are Huliu River, Yang River, Gui River and Qingshui River.

At present, the quality of Yongding River is the second good one in Beijing among all the natural water bodies. The sediment samples were taken from the Rearl Reservoir of the Yongding River.

- **Pearl River**

The Pearl River Delta which is located in the central coast of Guangdong Province with an area of 8,033 km^2 is the second largest estuarine delta in China after the Yangtze River Delta (supplementary Fig. S19). At present, there are more than 300 reaches in the river network of the Pearl River Delta with a total length of about 1,600 km and a drainage density as high as 0.81 km km^{-2}. The silt and clay consist of suspended sediments mainly. The runoff and sediment discharge of the Pearl River Delta varies greatly within a year because of the influence of the southern subtropical marine monsoon climate. More specifically, the runoff and sediment discharge during flood season (April-September) account for 74-84 % and 91-95 % of the total annual amount, respectively. The tide in the estuary of the Pearl River which is an irregular semidiurnal tide is small, with the average of 0.86 to 1.6 m and the maximum of 2.29 to 3.36 m.

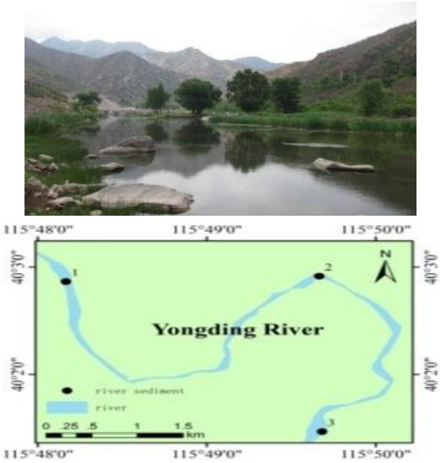

Figure S18. Landscape and geographical distribution of sampling sites in Yongding River. The photograph was taken by author Guibing Zhu. The map were come from web of "Data Sharing Infrastructure of Earth System Science" http://www.geodata.cn. All of the maps used in the manuscript are free. The geographical distribution of sampling sites was drawn with software ArcGIS.

Generally the drought period of Pearl River is from October to March of the next year. The annual average runoff is 80.3 billion m^3, only accounting for about 24 % of total annual basin runoff.

The sampling site of riparian zone (E 120°41'54.7", N 30°45'51.4") is close to Foshan city, Guangdong province. Three samples sites with intervals of 3 km were selected, and in each site three surface sediments (0-5 cm depth, 1 m away from the water-land interface) were collected from each plot in June and December 2011, respectively.

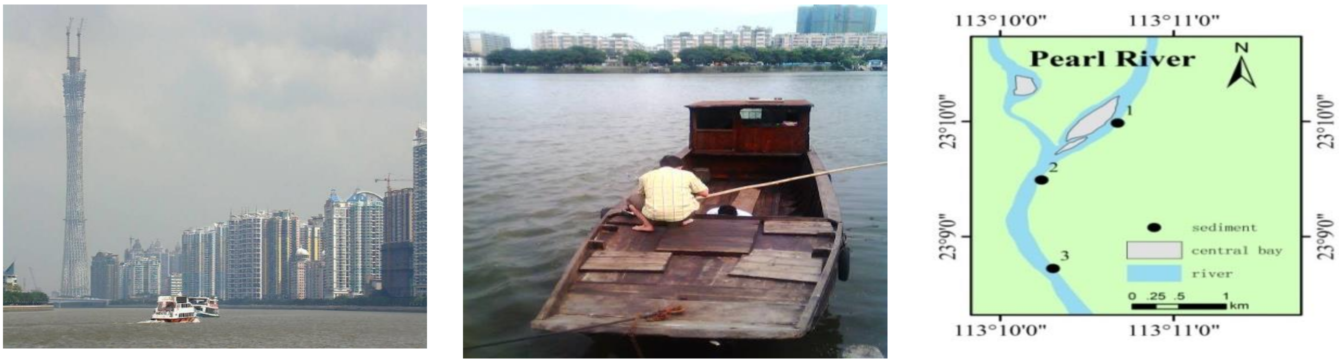

Figure S19. Landscape and geographical distribution of the sampling site and the author Dr. Guibing Zhu in Pearl River. The photograph was taken by author Shanyun Wang with the permission of Dr. Zhu. The map were come from web of "Data Sharing Infrastructure of Earth System Science" http://www.geodata.cn. All of the maps used in the manuscript are free. The geographical distribution of sampling sites was drawn with software ArcGIS.

- **Shahe reservoir**

Shahe reservoir is located in Changping district of Beijing City (supplementary Fig. S20). The reservoir received wastewater from neighbourhood and the water quality was not able to reach the standard of drinking water source. In recent years, a series of actions were taken such as dredging, construction of wetland park, planting hydrophytes and establishment of wastewater treatment plants. Now, the water quality is improved greatly and the ecology of the reservoir shows its vitality that a large amount of migrant birds like black swans gathered here.

Core samples were taken from the littoral zone of the reservoir.

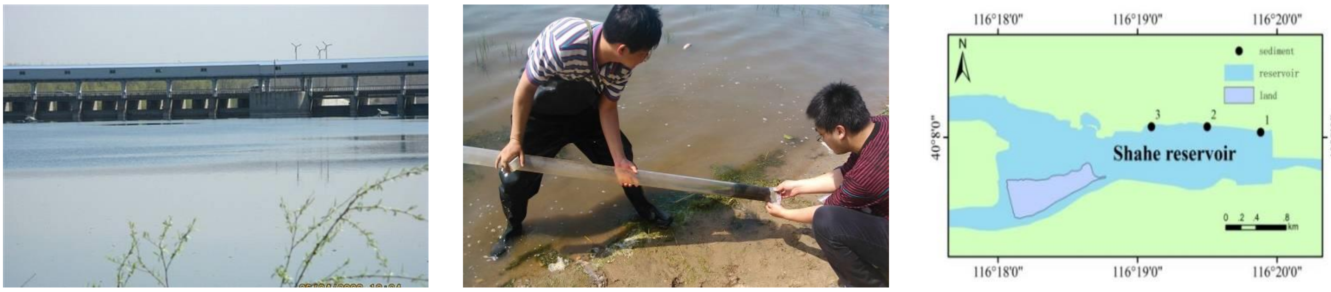

Figure S20. Landscape and geographical distribution of the sampling site and the author Dr. Guibing Zhu and Bo Jiang in Shahe reservoir. The photograph was taken by author Leiliu Zhou with the permission of Zhu and Jiang. The map were come from web of "Data Sharing Infrastructure of Earth System Science" http://www.geodata.cn. All of the maps used in the manuscript are free. The geographical distribution of sampling sites was drawn with software ArcGIS.

- **Shangqiu Zhengge reservoir**

Shangqiu city is located in the warm temperate zone with a semi-moist continental monsoon climate (supplementary Fig. S21). The climate is characteristic of strong wind in spring, hot and rainy in summer, cool and long sunshine in autumn and cold with little rain and snow in winter. The annual average sunshine hours are 1,944 and the frost-free period is about 211 days. The annual average temperature is 14.2 ℃ ranging from 39 to -9 ℃. Average annual precipitation is 623 mm. the total area of Zhengge reservoir 15.3 km^2. Total storage capacity of the reservoir is 26.3 million m^3 which is mainly from Yellow River. The reservoir was officially used as part of the drinking water source for Shangqiu city since 2004.

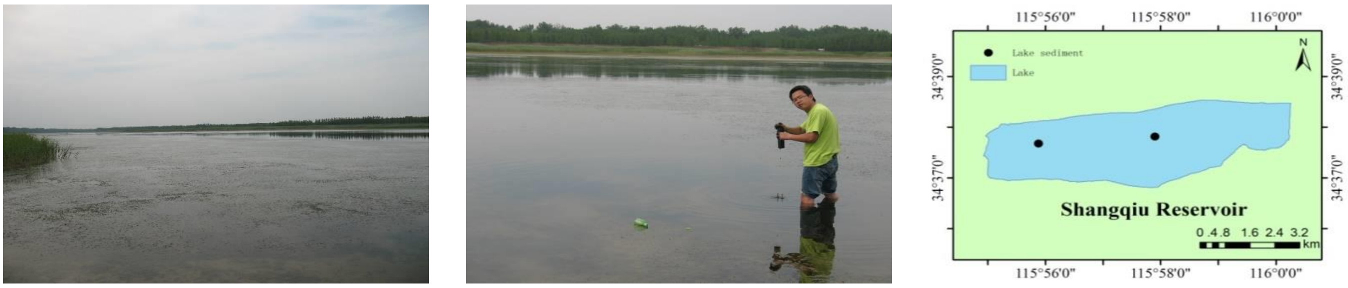

Figure S21. Landscape and geographical distribution of the sampling site and the author Dr. Yu Wang in reservoir. The photograph was taken by author Guibing Zhu with the permission of Dr. Wang. The map were come from web of "Data Sharing Infrastructure of Earth System Science" http://www.geodata.cn. All of the maps used in the manuscript are free. The geographical distribution of sampling sites was drawn with software ArcGIS.

- **Jiaxing paddy soils**

The long-term fertilized paddy soil used in this study is located at Meilin Town, Jiaxing City, Zhejiang Province, China (E 120°41’54.7”, N 30°45’51.4”) (supplementary Fig. S22). This site represents a typical agricultural region of subtropical China. It has a subtropical monsoon climate with an annual rainfall of 1,300 mm and annual average temperature of 18 ℃.

The Jiaxing paddy soil was planted with rice twice in February and October every year with the practice of feeding livestock waste for more than 25 years. A long-term fertilizer practices was fed with livestock waste including urine, dung and flushing water which was primarily fermented and stored in the fermentation tank to fertilize the paddy soil periodically. The fertilized wastewater was characteristic of high ammonia, total nitrogen and chemical oxygen demand. The soil is classified as agri-udic ferrosols with a silty clay texture (clay 40.0%, silt 55.0% and sand 5.0%) derived from quaternary clay earth.

The soil samples were collected in November (autumn) 2008 at depth from 0 to -100 cm. Three soil cores (approximately 5 cm diameter) with different depths (every 10 cm) were taken from each plot and mixed to form one composite sample.

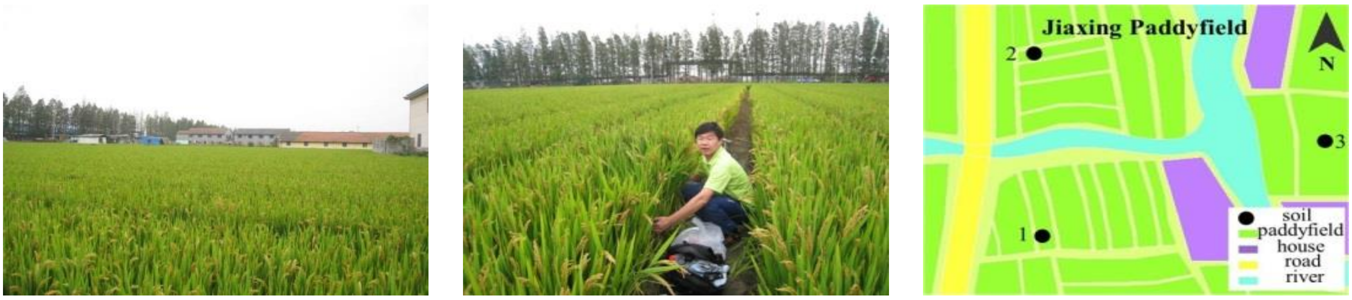

Figure S22. Landscape and geographical distribution of the sampling site and the author Dr. Guibing Zhu in Jiaxing paddy field. The photograph was taken by author Guibing Zhu with remote control. The map were come from web of “Data Sharing Infrastructure of Earth System Science” http://www.geodata.cn. All of the maps used in the manuscript are free. The geographical distribution of sampling sites was drawn with software ArcGIS.

- **North Canal**

The length of the main stream of North Canal is 142.7 km, with the average width of 80 - 100 m (supplementary Fig. S23). There is Wenyu River in the upper reach, which meets with Tonghui River when comes to Tongxian county, and finally empties into Haihe River. According to Darcy’s law, the degree of exchange of surface water and groundwater depends on hydraulic gradient and hydraulic conductivity. North

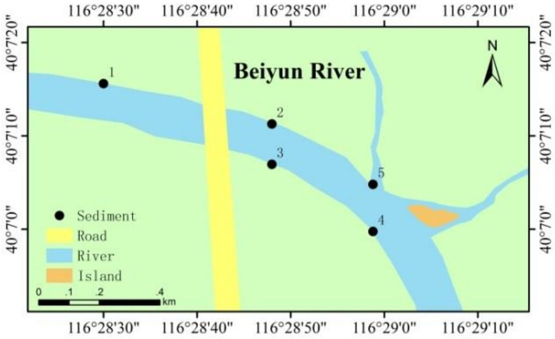

Figure S23 Geographical distribution of the sampling site in North Canal drawn with software ArcGIS.

canal has large time gradient of water level because it undergoes drought and flood periods intermittently, and the construction of numerous dams promotes the violent interaction of surface water and groundwater. With the rapid developing of Beijing, North Canal has turned to be the major sewage river because the rivers from the suburban areas of Beijing all empty into North Canal, resulting in serious pollution in the river and the water quality along the major water body is worse than Grade V, the worst water quality standard for surface water in China. The complex water environment, wide area of floodplain, high heterogeneity of landscape and great biodiversity make this area to be a ideal experimental field for the study of anammox.

- **Ningxia paddyfield**

The paddy field is located at 35 °14' - 39 °23' N and 104 °17' - 107 °39' E in Ningxia Hui Autonomous Region with an altitude of 1100 to 1200 m (supplementary Fig. S24). The Region is far from the ocean and the climate varies greatly between the north and south edge. The southern part of Ningxia locates in the semiarid region of the south temperate zone, while the northern and central part locates in the arid and semiarid region of the temperate zone, respectively. The annual average temperature is 5 to 9 ℃ and the precipitation is mostly occurred in summer. The south part normally receives more precipitation.

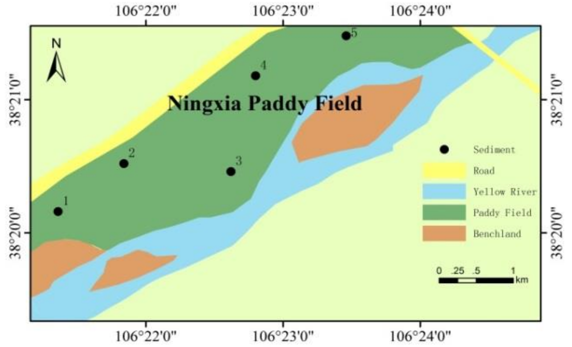

Figure S24 Geographical distribution of sampling sites in the Ningxia paddy field drawn with software ArcGIS.

There are more than 110 days when the daily average temperature is higher than 10 ℃ and more than 30 days when it is more than 18 ℃ in one year. The paddy field is irrigated with the water from Yellow River. The abundant water source and high water quality ensure the area an ideal place for the rice planting.

- **Changbai Mountain peatland**

The sampling site of peat wetland in Changbai Mountain (41°35′-42 °25'N; 127 °40'-128 °16'E) is located in the Antu County of Yanbian Prefecture, southeastern corner of the Jilin Province (supplementary Fig. S25). Changbai Mountain has a temperate continental and mountain climate. It is characteristic of long and cold winter, short and cool summer, windy spring and foggy autumn. The annual average temperature is -7 to 3 ℃. The Changbai

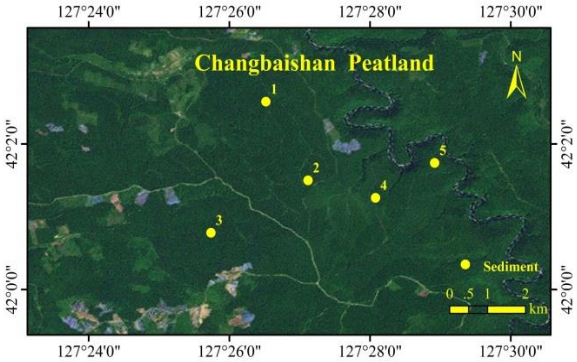

Figure S25 Geographical distribution of the sampling site in Changbai Mountain peatland drawn with software ArcGIS

Mountain has a varied natural environment and a changeable weather. The high biodiversity indicates an intergrated ecosystem of the Mountain. Three climatic zones including middle temperature zone, cold temperate zone and alpine frigid zone are formed with the rising of altitude. Five vertical vegetational zone including laurel forest zone, mixed wood zone, coniferous forest zone, betula ermanii forest zone and alpine tundra zone are distributed from the foot to the top of the mountain with the height of 2000 m. Changbai Mountain is called an "eco green lung" in northeast China. The Tumenjiang River、Songhua River and Yalu River which are all originated in Changbai Mountain form the water network in northeast China with the annual runoff of 24 billion m^3. High soil mositure make the mountain a unique soil and landscape for peats wetland.

- **Jiamusi swamp**

The Jiamusi swamp (45°01′-48°27′ N; 130°13′-135°05′ E) is located in Sanjiang Plain in the northeastern corner of the Northeast China Plain (supplementary Fig. S26). The Jiamusi swamp is alluvial by three rivers of Heilongjiang River、Ussuri River and Songhua River and it is also the largest swamp distributed area in China.

The rainfall of Jiamusi swamp is mostly occurred in summer and autumn. The slow runing rivers and viscous soil with repeated process of freeze and thaw make the surface wet in most of the time. These are ideal conditions for the formation of swamp that the area of swamp and swamping land is as large as 2.4 million hectare.

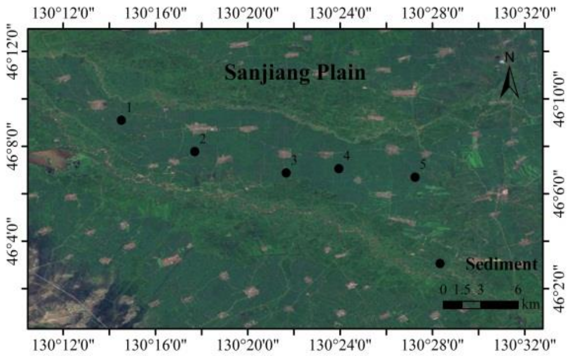

Figure S26 Geographical distribution of the sampling site in Jiamusi swamp in Sanjiang Plain drawn with software ArcGIS

Jiamusi swamp has a climate of humid or sub-humid continental monsoon in temperate zone. The annual sunshine time is 2400 to 2500 hours. The average temperature in January and July is -21to -18 ℃ and 21to 22 ℃, respectively. The annual accumulated temperature varies from 2300 to 2500 ℃. The frost-free period is 120 to 140 days and the frozen period is as long as 7 to 8 months with the maximum frozen depth of 1.5 to 2.1 m. The annual precipitation is 500 to 650 mm and 75 to 85 % of the them occur during June and October.

The main hygrophytes in Jiamusi swamp includes *Calamagrostis Angustifolia*, *Salix Brachypoda*, *Sedge* and *Phragmites Australis*. The *Sedge* is the dominant species, which covers 85 % area of the swamp. The second one is the *Phragmites Australis*. The soil types includes black soil, albic soil, meadow soil and boggy soil with the meadow soil and boggy soil is the most widely distributed types.

- **Yanbian paddy field**

Yanbian paddy field lies in the Yanbian Korean Autonomous Prefecture (41°59′ - 44°30′ N; 127°27′ - 131°18′ E), Jilin Province (supplementary Fig. S27). As a main rice producing area, there is a long history of rice planting in the Prefecture. The fecund soil, densely distributed rivers and adequate water resource make it an ideal place for rice planting.

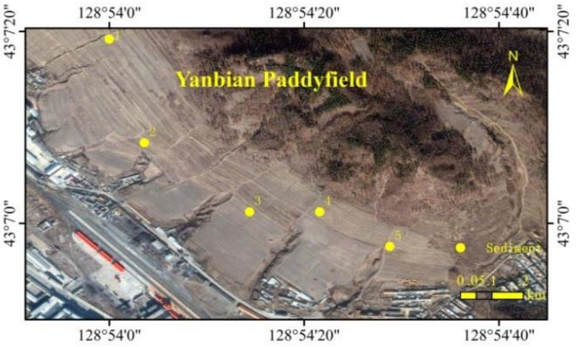

Figure S27 Geographical distribution of the sampling site in Yanbian paddy field drawn with software ArcGIS

The Prefecture has a temperate and humid monsoon climate. The annual sunshine time is 2300 to 2500 hours and the frost-free times is 100 to 150 days. The annual precipitation is 500 to 700 mm. The day and night temperature difference is more than 10 ℃ and the largest one is 17.4 ℃, which is favour to the formation of the dry matter in rice. So the rice here is often of high quality and nutrition.

- **Qinghai-Tibet Plateau swamp**

Qinghai-Tibet Plateau lies in 25°-40° N; 74-104°E. It is the highest plateau in the world with the average elevation higher than 4,000 m (supplementary Fig. S28). It is also called "The Roof of the World" or "The Third Pole". The Plateau is full of glaciers, alpine lakes and alpine swamps. Many important rivers in Asia are originated from the Plateau.

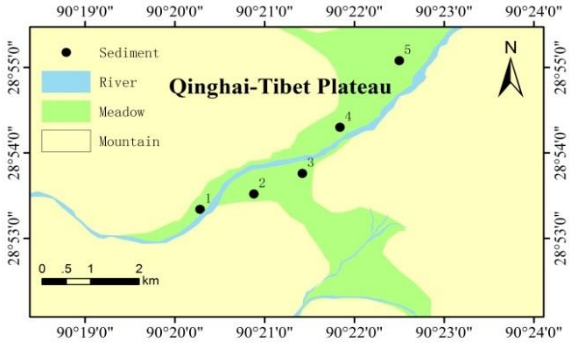

Figure S28 Geographical distribution of the sampling site in Qinghai-Tibet Plateau swamp drawn with software ArcGIS

The alpine swamps are widely distributed in Qinghai-Tibet Plateau. It is mostly caused by the plateau cold climate in the following three aspects:

1) The effect of evaporation is usually weak due to the low temperature in high altitude zone.
2) The abundant melt water accumulated in lowland.
3) Large area of impermeable layer was formed because of the frozen soil layers in the plateau.

As the highest swamp in the world, the average elevation of this area is 4,000 m with the highest point of 5,350 m. The annual temperature is 1-3 ℃ and the annual precipitation is 300-700 mm, which shows a cold and moist climate. The water resource is abundant with plenty of meltwater or fountains. High sunlight intensity and low temperature at night lead to high plant productivity and hard to decomposition in the swamp. Peat is easy to be accumulated in the swamp and turns into peat soil or peat mire soil. Plants that often appear in the north temperate zone are easy to be observed and the *Cyperaceae Kobresia Willd* controls the plant community in the swamp. According to preliminary

investigation, there are about 220 species (51 families and 101 genera) of higher plant distributed in this area.

- **Hunan paddy field**

The site is located at Yueyang Red Soil Experimental Station (26°45′N, 111°52′E), Hunan Province, China (supplementary Fig. S29). A long-term fertilizer experiment was established in 1990 with a wheat - maize rotation system. This site represents a typical agricultural region of subtropical China. It has a subtropical monsoon climate with an annual rainfall of 1300 mm and annual average temperature of 18 ℃. The soil is classified as agri-udic ferrosols with a silty clay texture (clay 45.0%, silt 46.3% and sand 8.7%) derived from quaternary red clay earth.

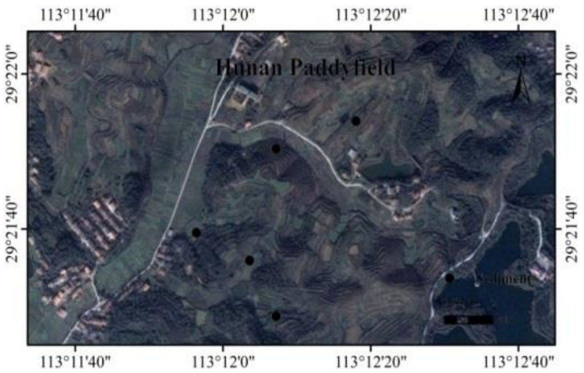

Figure S29 Geographical distribution of the sampling site in Yueyang paddy field drawn with software ArcGIS

- **Antu reservoir**

Antu reservoir which is located in Buerhatong River of Jilin Province is an artificial reservoir built in 1968 (supplementary Fig. S30). Impacted by the monsoon, it is hot and rainy in summer with the highest temperature at 36 ℃, whereas it is cold and dry in winter with the lowest temperature at -36 ℃ in this area. The regular capacity of the reservoir is 37.03 million m^3 with the catchment area of 370 km^2 and the height of 382 m. Antu reservoir is also the drinking water source of Mingyue Town, Antu County.

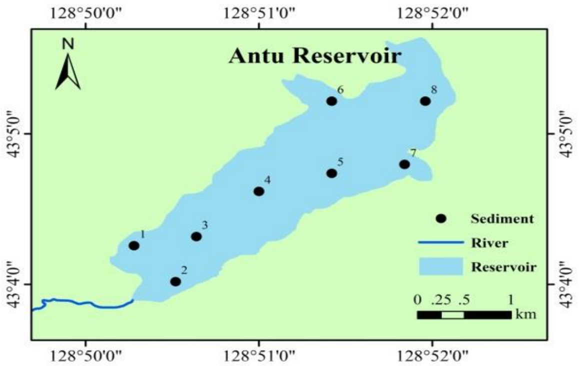

Figure S30 Geographical distribution of the sampling site in Antu reservoir drawn with software ArcGIS

- **Changshu paddy field**

Changshu City (31°33′-31°50′N; 120°33′-121°03′E) is located at the southeast of Jiangsu Province (supplementary Fig. S31). The city has a low and flat topography with the elevation of 3 to 7 m. Changshu lies in the middle latitude region and has a subtropical monsoon climate with a

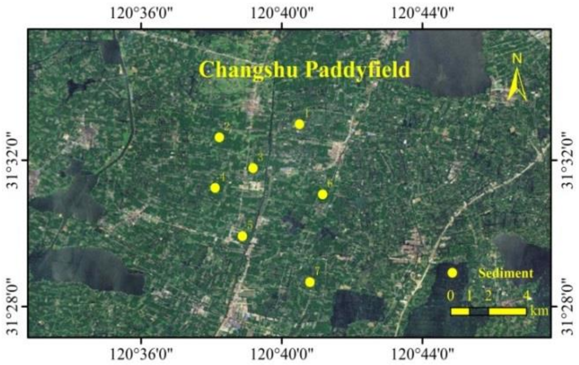

Figure S31 Geographical distribution of the sampling site in Changshu paddy field drawn with software ArcGIS

temperate climate, well-marked seasons and abundant rainfall. It is cold and rainless with the north wind from the continent in the winter. Whereas, it is hot and rainy with the wind from the southeast of ocean in the summer. The annual time of sunshine is 2130 hours which accounts for 48 % of the available sunshine hour. The annual average temperature is 15.3 ℃ and the annual precipitation is 1054 mm. The paddy field in Xinzhuang Town in the Taihu Lake Basin is selected as the sampling site.

- **Meihekou paddyfield**

Meihekou City (42-43 °N; 125-126 °E) is located at the southeast part of Jilin province (supplementary Fig. S32). It lies in the west of Changbai Mountains and the upstream of Huifa River. The city is also the interchange of Changbai Mountains and Songliao Plain. Meihekou City has a temperate continental monsoon climate and the annual average temperature is 4.6 ℃. The city has long been enjoying the prestige of "a city with a thousand reservior" and "a land flow with milk and honey" due to rich water resources. It is the national commodity grain base and the key producing area of high quality rice. There are 30 000 hm^2 of paddy field in Meihekou City where the eco-environment is very suitable for rice planting.

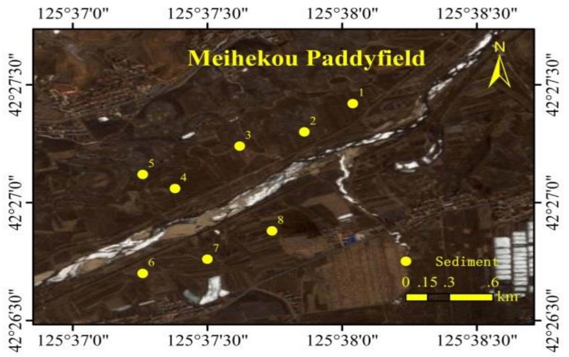

Figure S32 Geographical distribution of the sampling site in Meihekou paddy field drawn with software ArcGIS

- **Shimen swamps**

Shimen swamps (42°01′-43°24′N; 127°48′-129°11′E) is located at the southwest corner of the Yanbian Korean Autonomous Prefecture, Jilin Province (supplementary Fig. S33). The climate is characteristic of short growth period for vegetation, low temperature, abundant rainfall and sunshine. The famous Changbai Mountains is located in Antu County, so Antu is called “first county of Changbai Mountains”.

The natural resources are very abundant here. 88 rivers which are over 1,800 km long in total lies in Antu county. The annual runoff could reach 4 billion m^3. Most of the rivers are groundwater fed and flows all through the year.

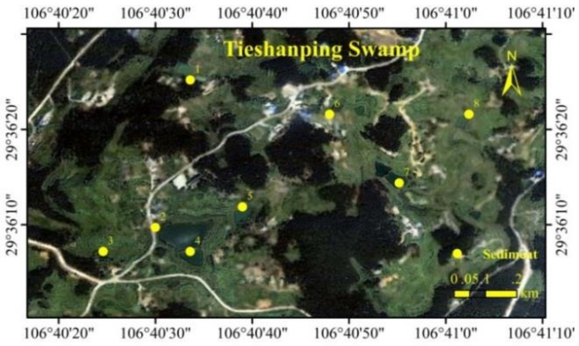

Figure S33 Geographical distribution of the sampling site in Shimen swamps drawn with software ArcGIS
